# Autophagy Correlates with the Therapeutic Responsiveness of Malignant Pleural Mesothelioma in 3D Models

**DOI:** 10.1371/journal.pone.0134825

**Published:** 2015-08-18

**Authors:** Dario Barbone, Carlo Follo, Nohemy Echeverry, Victor H. Gerbaudo, Astero Klabatsa, Raphael Bueno, Emanuela Felley-Bosco, V. Courtney Broaddus

**Affiliations:** 1 Division of Pulmonary and Critical Care Medicine, Department of Medicine, San Francisco General Hospital, University of California San Francisco, San Francisco, California, 94110, United States of America; 2 Clinic of Oncology, University Hospital Zurich, 8044 Zurich, Switzerland; 3 Division of Nuclear Medicine & Molecular Imaging, Department of Radiology, Brigham & Women's Hospital and Harvard Medical School, Boston, Massachusetts, 02115, United States of America; 4 Department of Research Oncology, King's College London, London, United Kingdom; 5 Department of Surgery, Brigham and Women's Hospital, Harvard Medical School, Boston, Massachusetts, 02115, United States of America; National Cheng Kung University, TAIWAN

## Abstract

Malignant pleural mesothelioma is a highly chemoresistant solid tumor. We have studied this apoptotic resistance using *in vitro* and *ex vivo* three-dimensional models, which acquire a high level of chemoresistance that can be reduced by PI3K/mTOR inhibitors. Here, we investigate the activity of GDC-0980, a novel dual PI3K/mTOR inhibitor, which has been proposed to be effective in mesothelioma. In this work, we aimed to identify mechanisms and markers of efficacy for GDC-0980 by utilizing 3D models of mesothelioma, both *in vitro* multicellular spheroids and *ex vivo* tumor fragment spheroids grown from patient tumor samples. We found that a subset of mesothelioma spheroids is sensitive to GDC-0980 alone and to its combination with chemotherapy. Unexpectedly, this sensitivity did not correlate with the activation of the Akt/mTOR pathway. Instead, sensitivity to GDC-0980 correlated with the presence of constitutive ATG13 puncta, a feature of autophagy, a cellular program that supports cells under stress. In tumor fragment spheroids grown from 21 tumors, we also found a subset (n = 11) that was sensitive to GDC-0980, a sensitivity that also correlated with the presence of ATG13 puncta. Interference with autophagy by siRNA of ATG7, an essential autophagic protein, increased the response to chemotherapy, but only in the sensitive multicellular spheroids. In the spheroids resistant to GDC-0980, autophagy appeared to play no role. In summary, we show that GDC-0980 is effective in mesothelioma 3D models that display ATG13 puncta, and that blockade of autophagy increases their response to chemotherapy. For the first time, we show a role for autophagy in the response to chemotherapy of 3D models of mesothelioma and propose ATG13 as a potential biomarker of the therapeutic responsiveness of mesothelioma.

## Introduction

Malignant pleural mesothelioma is a recalcitrant solid tumor of the pleural lining for which, to date, no curative therapy is available. Our group has focused on the study of three-dimensional (3D) models of mesothelioma, which exhibit a high 3D multicellular resistance that may model the clinically relevant resistance of the actual tumor. In earlier studies, we have proposed a role for the PI3K/Akt/mTOR pathway in 3D multicellular resistance [1, 2].

The PI3K/Akt and mTOR pathways are frequently activated in mesothelioma [3–6] and have roles in its pathogenesis [7], survival and progression [8, 9]. Activation of the Akt/mTOR pathway is detected in the majority of mesothelioma cell lines [9] and, in mouse models of mesothelioma, its inhibition has been shown to improve response to chemotherapy [10–12]. Nevertheless, no inhibitory approach has yielded therapeutic value in a clinical setting [13]. We have previously tested several inhibitors of the PI3K/Akt (LY294002 and wortmannin) and mTOR (rapamycin) pathways or both (PI-103)[14], by using 3D mesothelioma models grown from cell lines (*multicellular spheroids*) and actual tumor (*tumor fragment spheroids*) [1, 2]. Importantly, our work has shown that the activity of the inhibitors was evident only in a 3D setting, where we could identify an important role for the mTOR pathway. With the recent development of dual inhibitors, we wanted to investigate the therapeutic advances of these new compounds by testing them in our 3D models.

Here we have focused our studies on GDC-0980 [15, 16], a recently developed, orally available agent that has shown antineoplastic activity by targeting PI3K and the two mTOR kinase complexes, mTORC1 and mTORC2. GDC-0980 has also been shown to be effective in *in vitro* studies of mesothelioma, in combination with a MET inhibitor [12]. Moreover, GDC-0980, whose safety has been described [17], showed efficacy in patients with advanced solid tumors, including mesothelioma (http://goo.gl/J42fEa). GDC-0980 is now in a Phase-1B clinical trial to determine its safety and pharmacology when in combination with either paclitaxel and carboplatin (with or without bevacizumab) or pemetrexed plus cisplatin in patients with solid tumors (http://goo.gl/Ck2vcN).

One aspect of 3D biology that is beginning to be explored and might have relevance when studying PI3K/Akt/mTOR inhibition is autophagy. With a survival role for cells under nutritional and pharmaceutical stresses, autophagy determines the fate of damaged organelles and cells through discrete, but overlapping, lysosomal and apoptotic pathways. While still under extensive investigation, autophagy may play important roles in both tumorigenesis and chemoresistance. The mTOR pathway represses autophagy and thus an inhibition of mTOR can activate autophagy, potentially altering the response of spheroids to chemotherapy. Indeed, GDC-0980 has been reported, like other PI3K/mTOR inhibitors [18], to induce autophagy [19]; nonetheless, little is known about how autophagy is altered by GDC-0980 in 3D. We considered that investigating the role of autophagy in a 3D setting and the effect of PI3K/mTOR inhibitors on autophagy would offer novel and potentially important clinical insights.

In the present work, we demonstrate that GDC-0980 is active in a subset of mesotheliomas, regardless of the activation of the Akt/mTOR pathway. In this sensitive subset, autophagy is induced by GDC-0980 and its inhibition further sensitizes these spheroids to chemotherapy. We found that the response to GDC-0980 correlated with the presence of ATG13 puncta, a marker of early autophagy. These novel findings in in vitro and ex vivo 3D models identify autophagy as a potential therapeutic target in a subset of tumors and suggest that markers of autophagy, such as ATG13 puncta, can be used to identify tumors sensitive to PI3K/mTOR inhibition.

## Material and Methods

### Cell culture and reagents

The human mesothelioma cell lines M28 [20] and VAMT [21] (both from Dr. Brenda Gerwin, NCI, National Institutes of Health, Bethesda, MD, USA), REN [22] (from Dr. Roy Smythe, University of Texas M.D. Anderson Cancer Center, Houston, TX, USA), SARC [23] (MesoSA1 – from Dr. Alice Boylan, Medical University of South Carolina, Charleston, MC, USA), JMN [24] and MSTO-211H [ATCC CRL-2081] (both from Dr. Dean Fennell, University of Leicester, UK) were all cultured in Dulbecco’s modified Eagle’s medium (DMEM) supplemented with 10% fetal bovine serum in a 37°C humidified incubator with 5% CO_2_. All were found to stain positively for mesothelioma markers (calretinin, WT1) and negatively for other markers not seen in mesothelioma (TTF1). Normal human mesothelial cells were cultured from ascites fluid from unidentified patients without infection or malignancy according to a protocol (#12–08998) approved by the University of California, San Francisco Committee on Human Research under our BUA (BU031309-03) [25]. All cells were confirmed to be negative for mycoplasma every 2 months by PCR analysis as previously described [26]. Bortezomib (25 nM), GDC-0980 (1 μM) and vorinostat (5 μM) were from Selleck Chem (Boston, MA, USA). Cisplatin (200 μM) and pemetrexed (10 μM) were obtained from the University of California, San Francisco Pharmacy at Mt. Zion Cancer Center.

### Generation and treatment of spheroids

Multicellular and tumor fragment spheroids were generated as previously described [27]. In brief, multicellular spheroids were generated as follows: 10^4^ cells were added to each well of a round-bottomed 96-well plate coated with polyHEMA (# P3932 Sigma-Aldrich, St. Louis, MO—5 mg/ml final concentration—1:24 dilution in 95% EtOH of a 120mg/ml stock solution also in 95% EtOH). After centrifugation for 5 minutes at 800 rpm, the spheroids were incubated at 37°C for 24h before harvesting.

Tumor fragment spheroids were generated as previously described [2] from 21 tumor samples obtained from extrapleural pneumonectomy (EPP) or pleurectomy procedures performed by R.B. at Brigham and Women’s Hospital in Boston, MA USA (additional data in S1 Table). Studies using human samples and de-identified data were carried out under an active University of California San Francisco CHR approval (#12–08998). Tissue was diced finely with scalpels to pieces smaller than 1 mm in diameter that were suspended in medium in 10 cm plates coated with 0.8% agar (Agar Noble, #A5431 Sigma-Aldrich, St. Louis, MO) in full DMEM. Media was changed twice a week and spheroids were utilized within 4 weeks of culture. Spheroids were cultured and treated in full DMEM as described in figure legends. An appropriate dilution of DMSO was used as vehicle control.

### Immunoblotting

After treatment, spheroids were lysed in cold RIPA buffer (NONIDET-P40 1%, NaDOC 0.5%, SDS 1%, in PBS). The concentration of total protein was evaluated with a colorimetric assay (DC protein assay from Bio-Rad, Hercules, CA, USA). 25 μg of cell lysates were loaded in reducing conditions (0.2 M Tris, pH 6.8, 5% SDS, 3% glycerol, 0.01% bromophenol blue and 200 mM DTT). After separation in SDS-PAGE (7.5 to 15% acrylamide) and transfer to PVDF (Immobilon, Millipore, Billerica, MA, USA), membranes were blocked with a protein-free TBS blocking buffer (Pierce, Rockford, IL, USA) and gently agitated with antibodies diluted in 5% non-fat dry milk or 5% BSA, as appropriate, at 4°C overnight. Secondary antibodies were from Amersham (Piscataway, NJ, USA). Chemiluminescence was detected by the enhanced SuperSignal West Pico Substrate (Pierce, Rockford, IL, USA) with a Biospectrum 810 imaging system (UVP, Upland, CA, USA). LC3 (#L7543) and tubulin (#T-6074) antibodies were from Sigma-Aldrich (St. Louis, MO, USA). P-AKT^Ser473^ (#4060), P-S6K^Thr389^ (#9234), P-ERK (#4370), Bid (#2002), ATG5 (#12994), ATG7 (#8558) and cleaved caspase 3 (#9661) antibodies were from Cell Signaling (Danver MA, USA). Bim (#559685) antibody was from BD Pharmingen (San Jose, CA, USA). Densitometry analysis was performed with VisionWorks software (UVP, Upland, CA, USA).

### RNA interference

Transient knockdowns were performed using Lipofectamine RNAiMAX according to the manufacturer’s protocol (Life Technologies, Carlsbad, CA, USA). In brief, cells were transfected in Opti-MEM (Life Technologies, Carlsbad, CA, USA) and allowed to grow as monolayers for 24 h in complete DMEM medium. Cells were then trypsinized, counted, plated in polyHEMA plates and allowed to form spheroids for 24 h. Spheroids were then treated as described in figure legends, between 48 and 72 h from transfection. Control (12935–400), Bim (s195011), Bid (s1986), ATG5 (HSS114104) and ATG7 (s20651) siRNA were purchased from Life Technologies (Carlsbad, CA, USA).

### Caspase 3/7 activation

Cleavage of caspases 3 and 7 was measured by the CaspaseGlo 3/7 assay kit (Promega, Madison WI). Briefly, spheroids were treated as indicated in the polyHEMA-coated 96 wells in which they were grown (200 μl final volume). One spheroid was used for each condition; studies were performed in quadruplicate. After treatment, plates were spun at 400 g for 10 min RT; 100 μl of supernatant were carefully removed without disturbing the pellet and 100 μl of complete CaspaseGlo 3/7 reagent was added to each well. Plates were gently mixed using a plate shaker at 300–500 rpm for 1 min and then incubated at RT away from light for 1 h. Sample luminescence was measured in a plate-reading luminometer (Perkin Elmer, Waltham, MA).

### Nuclear condensation assay using Hoechst staining

Monolayers and spheroids were disaggregated with trypsin for the same period of time, washed with ice-cold PBS, and then fixed with 2.5% glutaraldehyde (Sigma-Aldrich, St. Louis, MO, USA). Cells were then stained with 8 μg/ml of Hoechst 33342 (Molecular Probes, Life Technologies, Carlsbad, CA, USA) and placed on slides. Apoptosis was quantitated by counting cells with distinctive signs of nuclear condensation and expressed as a % of the total cells. For each condition, at least 300 cells were counted in triplicate by investigators blinded to the experimental conditions. We have found that this assay is more accurate than assays that detect surface phosphatidylserine in cells disaggregated from spheroids [1]. Cells showing an apoptotic response both to GDC-0980 alone (compared to control) and to the combination of GDC-0980 plus chemotherapy (compared to chemotherapy alone) were considered *sensitive* to GDC-0980.

### Cleaved Caspase 3 staining in tumor fragment spheroids

Measurement of apoptosis in tumor tissue required dual immunostaining to identify the mesothelioma cells and then to localize active caspase 3 to mesothelioma cells, as we have previously reported [18]. Following treatment, tumor fragment spheroids were collected, fixed in 10% buffered formalin in PBS overnight at 4°C and embedded in 3% agar in PBS. The agar pellets containing the tumor fragment spheroids were further embedded in paraffin blocks by the University of California, San Francisco pathology core at San Francisco General Hospital. 5 μm paraffin sections were deparaffinized with xylene (2x5 min), 100% EtOH (2x2 min), 95% EtOH (2x2 min), 70% EtOH (2x2 min), 50% EtOH (1x2 min) and ddH20 (2x2 min). Antigens were retrieved in citrate buffer (Citra, BioGenex; #HK087-5K) in a pressure cooker at the highest setting for 10 min. Sections were blocked with 5% BSA and 0.1% Tween in PBS for 45 min in a humidified chamber at RT. Primary antibodies for cleaved caspase-3 (rabbit polyclonal 1:100–#9661 – Cell Signaling, Danvers, MA, USA) and pan-cytokeratin (mouse monoclonal 1:200—M3515 clone AE1/AE3, DAKO, Carpinteria, CA, USA) were incubated in a humidified chamber at 4°C overnight. The secondary antibodies for *cleaved caspase 3* (#A11010 goat anti-rabbit AlexaFluor 546, Life Technologies, Carlsbad, CA, USA) and *cytokeratin* (RPN1001V biotinylated sheep anti-mouse, GE Healthcare and #A6374 NeutrAvidin streptavidin-conjugated oregon green–Life Technologies, Carlsbad, CA) both 1:200, were incubated for 60 min at RT in a humidified chamber. Slides were washed 3 times in PBS-Tween for 3 min and mounted with ProLong Gold antifade reagent (#P36930, Life Technologies, Carlsbad, CA, USA). Slides were allowed to cure overnight at RT and were then imaged and photographed using a Nikon C1 confocal microscope. In a blinded fashion, the investigators examined captured images of doubly stained cells in the tumor fragment spheroids. Apoptotic mesothelioma cells were considered to be cells with merged red (pan-cytokeratin) and green (cleaved caspase 3) and were expressed as a percentage of the total number of mesothelioma cells (red). For each condition, 3–10 spheroids were counted until a total of 300 mesothelioma cells were visualized. Spheroids with an apoptotic response both to GDC-0980 alone (compared to control) and to the combination of GDC-0980 plus chemotherapy (compared to chemotherapy alone) were considered *sensitive* to GDC-0980.

### ATG13 Immunofluorescence

Multicellular spheroids were disaggregated with trypsin and 2 x 10^4^ cells were cytospun on glass slides. Slides with adhered cells were fixed in paraformaldehyde 4% in TBS overnight at 4°C then washed 3 times in TBS-Tween (0.1%), saturated in TBS/BSA 1% for 1 h and washed again 3 times in TBS-Tween 0.1%. Slides were then incubated overnight with an ATG13 antibody (1:100, #13468, Cell Signaling, Danvers, MA, USA), then washed 3 times in TBS-Tween and incubated 1.5 h at RT with a goat anti-rabbit-AlexaFluor 546 secondary antibody (#A11010, Life Technologies, Carlsbad, CA, USA). Slides were then washed 3 times in TBS-Tween, incubated with TOPRO-3 for 30 min at RT, washed again 3 times in TBS-Tween and mounted on slides with ProLong Gold antifade reagent (#P36930, Life Technologies, Carlsbad, CA, USA). Slides were allowed to cure overnight at RT and were then imaged and photographed using a Nikon C1 confocal microscope and examined for the presence of bright aggregates of ATG13.

Tumor fragment spheroids were processed as for cleaved caspase 3 staining described above. Primary antibodies for ATG13 (1:100, #13468, Cell signaling, Danvers, MA, USA) and pan-cytokeratin (1:200—M3515 clone AE1/AE3, DAKO, Carpinteria, CA, USA) were incubated in a humidified chamber at 4°C overnight. The secondary antibodies for ATG13 (#A11010 goat anti-rabbit AlexaFluor 546, Life Technologies, Carlsbad, CA, USA) and cytokeratin (#RPN1001V biotinylated sheep anti-mouse, GE Healthcare and #A6374 NeutrAvidin streptavidin-conjugated Oregon green–Life Technologies, Carlsbad, CA) both 1:200, were incubated for 60 min at RT in a humidified chamber. All slides were allowed to cure overnight at RT and were then imaged and photographed using a Nikon C1 confocal microscope. Mesothelioma cells were identified by their cytokeratin staining. Tumor fragment spheroids with more than 10% of mesothelioma cells with ATG13 puncta were considered positive.

### Immunohistochemistry

Tumor fragment spheroids used for the apoptosis studies were probed for expression of Bim, Akt, P-Akt, P-S6K, Ki67, GLUT-1 and IRS-1 protein levels by immunohistochemistry. Paraffin sections (5 μm) were stained for 2 h at RT with antibodies for Bim (1:200 #559685BD, Pharmingen, San Jose, CA), P-AKT^Ser473^ and P-S6K^Thr389^ (1:100 #4060, 1:100 #9234, Cell Signaling, Danver MA, USA), Ki67 (1:200, #ab15580, Abcam, Cambridge, MA, USA), GLUT-1 and IRS-1 (1:500 PA5-16793, 1:800 PA5-29667, Thermo Scientific, Waltham, MA, USA) and visualized with a HRP/DAB Envision plus Kit (#K4010, Dako, Carpinteria, CA). Known negative and positive controls were included. Hematoxylin was used as a counterstain. Slides were viewed by two independent scorers and scored semi-quantitatively (0 = no staining, 1 = weak staining in 0–25% cells, 2 = moderate staining in 0–50% of cells, 3 = strong staining in 50–100% of cells).

### FDG-PET/CT Imaging and Image Analysis

Tumor fragment spheroids were grown from tumor resected from patients who had undergone FDG-PET/CT imaging for initial staging of their disease and the data was provided without identifiers. The FDG-PET/CT was performed as follows. After fasting for a period of 6 h, patients were injected intravenously with 12 mCi of ^18^F-fluorodeoxyglucose (FDG). Following an uptake phase of 60 ± 10 min post-injection, they were scanned with a combined PET/CT scanner (Discovery ST; General Electric Healthcare, Waukesha, WI, USA). Scans were acquired in patients in the supine position with their arms placed above their heads when possible, and without any specific breath-holding instructions. Unenhanced CT scans for attenuation correction and anatomic co-registration were performed first, from the patient’s head to the mid thighs, using the following acquisition parameters: 140kVp, 75–120mA (varying according to the patient’s weight), 0.5 sec per CT rotation, a pitch of 1.375:1, and a reconstructed slice thickness of 3.75 mm. FDG-PET emission scans were acquired in 3-dimensional mode starting at the mid-thighs toward the head, for 6 to 7 bed positions of 3 minutes each. The CT data were reconstructed using a filtered back projection algorithm. PET data were reconstructed using an ordered-subset expectation maximization iterative algorithm (28 subsets, 2 iterations), yielding a volume of 47 slices. Metabolic tumor volume analysis in the FDG-PET images was done on a dedicated nuclear medicine workstation (Hermes, Stockholm, Sweden). Primary mesothelioma lesions’ metabolic tumor burden was calculated with a semi-automatically defined volume of interest using the mean intensity of the liver plus 2 SD as the threshold. Briefly, after the threshold was defined, the software generated 3D tumor regions from which measures of the total tumor metabolic volume (*MTV*), maximum standardized uptake value (*SUVmax*) and averaged SUV of the entire tumor (*SUVavg*) were obtained. In some cases, manual adjustments of the estimated tumor boundaries were applied by the operator to avoid overestimation of the metabolic tumor volume. Then, total lesion glycolysis (*TLG*) in mesothelioma lesions was calculated by multiplying the SUVavg by the MTV.

### Human subjects research

University of California San Francisco CHR approved the studies on human samples under an exempt category (#12–08998) reviewed by the University of California San Francisco Committee on Human Research, Laurel Heights Committee (CHR FWA number: 00000068 IRB registration number: 00003471). Exemption was granted because the "research involves the collection or study of existing data, documents, records, pathological specimens, or diagnostic specimens" and because "the information is recorded by the investigator in such a manner that subjects cannot be identified, directly or through identifiers linked to the subjects". Both of these statements were met in our research. Moreover, "the coded private information or specimens were not collected specifically for the current proposed research project and the key to decipher the code is destroyed before researcher begins". Samples were obtained from a tissue bank managed by Dr. Raphael Bueno, Chief, Division of Thoracic Surgery at Brigham and Women's Hospital, Boston MA. The University of California, San Francisco CHR committee approved the use of these unidentified mesothelioma samples for our research. Normal mesothelial cells were obtained under the same CHR-exempt authorization from unidentified patients' material (ascites or pleural fluid) that would otherwise be discarded

### Statistical analysis

Data are expressed as mean ± standard deviation or standard error of the mean (as appropriate) of at least three different experiments. Statistical significance was evaluated by ANOVA (with Tukey’s test to determine where the difference lay) or by linear correlation analysis to calculate a Pearson correlation coefficient (GraphPad Prism v 4.0, GraphPad Software, Inc., La Jolla, CA, USA). A *p* value *≤* 0.05 was considered significant.

## Results

### GDC-0980 is effective in a subset of 3D multicellular spheroids

**Fig 1:**
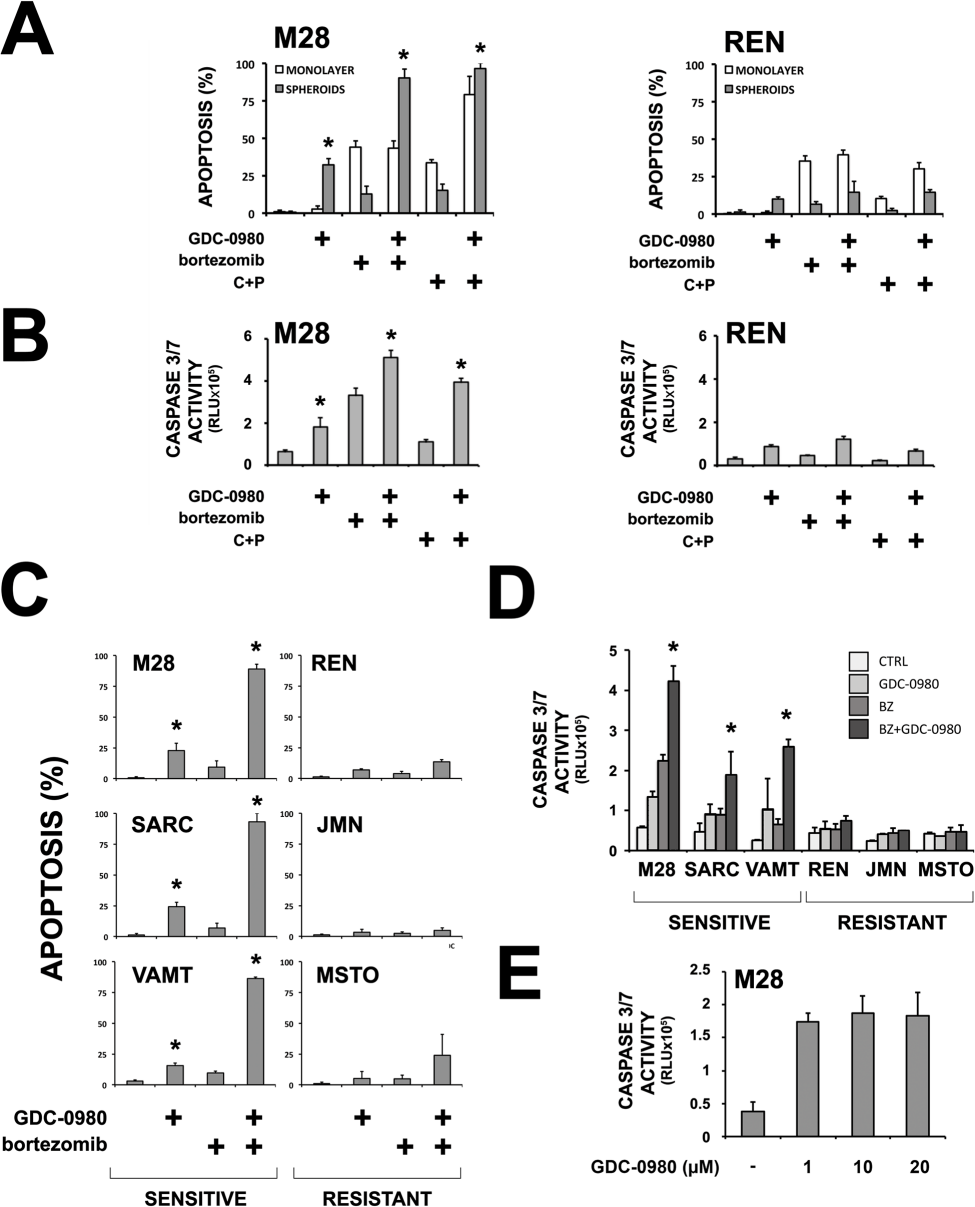
A subset of mesothelioma cells is sensitive to GDC-0980. (A) M28 and REN monolayers and spheroids were treated with GDC-0980 (1 μM), bortezomib (25 nM), cisplatin (200 μM) plus pemetrexed (10 μM)(C+P) or the combinations for 24 h. Cells with condensed nuclei seen after Hoechst staining were considered apoptotic (* p < *0*.*05* compared to the same treatment without GDC-0980; *n* = 3; mean ± SD). In the spheroids, GDC-0980 had activity when given alone and significantly potentiated chemotherapy in M28 cells but not in REN cells. (B) CaspaseGlo on M28 and REN spheroids treated as previously for 16 h. Caspase 3/7 activation is expressed as relative luminescence units (RLUx10^5^). Again, M28 cells demonstrated sensitivity to GDC-0980 and REN cells demonstrated resistance. (* p < *0*.*05* compared to the same treatment without GDC-0980; *n* = 3; mean ± SD). (C) Six cell lines (M28, SARC, VAMT, REN, JMN, MSTO-211H) grown as spheroids were treated with GDC-0980 (1 μM), bortezomib (25 nM) or the combination for 24 h. Three cell lines showed a response to GDC-0980 (*sensitive*) and 3 showed no response (*resistant*). (* p < *0*.*05* compared to the same treatment without GDC-0980; *n* = 3; mean ± SD) (D) Caspase 3/7 activation in spheroids treated similarly for 16 h, measured by CaspaseGlo, confirmed the differences between the sensitive and resistant spheroids. (* p < *0*.*05* different from the response to chemotherapy alone; *n* = 3; mean ± SD) (E) M28 spheroids were treated with higher concentrations of GDC-0980 (1,10 and 20 μM) for 24 h and caspase 3/7 activity was measured by CaspaseGlo. Higher concentrations of GDC-0980 did not increase its efficacy.

To test the activity of GDC-0980, we used monolayers and multicellular spheroids grown from two mesothelioma cells lines routinely used in our lab (M28 and REN). We treated monolayers and spheroids with GDC-0980 alone or in combination with the current standard of therapy for mesothelioma, cisplatin plus pemetrexed, or with a proteasome inhibitor, bortezomib [1, 27–30]. Interestingly, GDC-0980 was active only in the M28 cells, either when used alone or in combination with bortezomib or cisplatin plus pemetrexed (Fig 1A). In REN cells, however, GDC-0980 did not show activity in either monolayers or spheroids. We confirmed these results by measuring the activation of caspase 3 and 7 with a CaspaseGlo assay (Fig 1B). To investigate if sensitivity to GDC-0980 is a feature of a subset of mesotheliomas, we studied 4 more mesothelioma cell lines (SARC, VAMT, JMN and MSTO-211H). Using 3D multicellular spheroids with and without bortezomib, we found two distinct groups: one group was sensitive (M28, SARC, VAMT) and the other, resistant (REN, JMN, MSTO-211H) to GDC-0980 (Fig 1C and 1D). Of importance, GDC-0980 had little to no activity in normal mesothelial cells (S1 Fig). Wallin *et al*. have shown that GDC-0980 has broad activity in the range of 500 nM [15] on a panel of 167 tumor cell lines. In our experiments, we used a concentration of 1 μM; higher concentrations (10 and 20 μM) did not improve the efficacy of GDC-0980 (Fig 1E—M28 spheroids).

**Fig 2:**
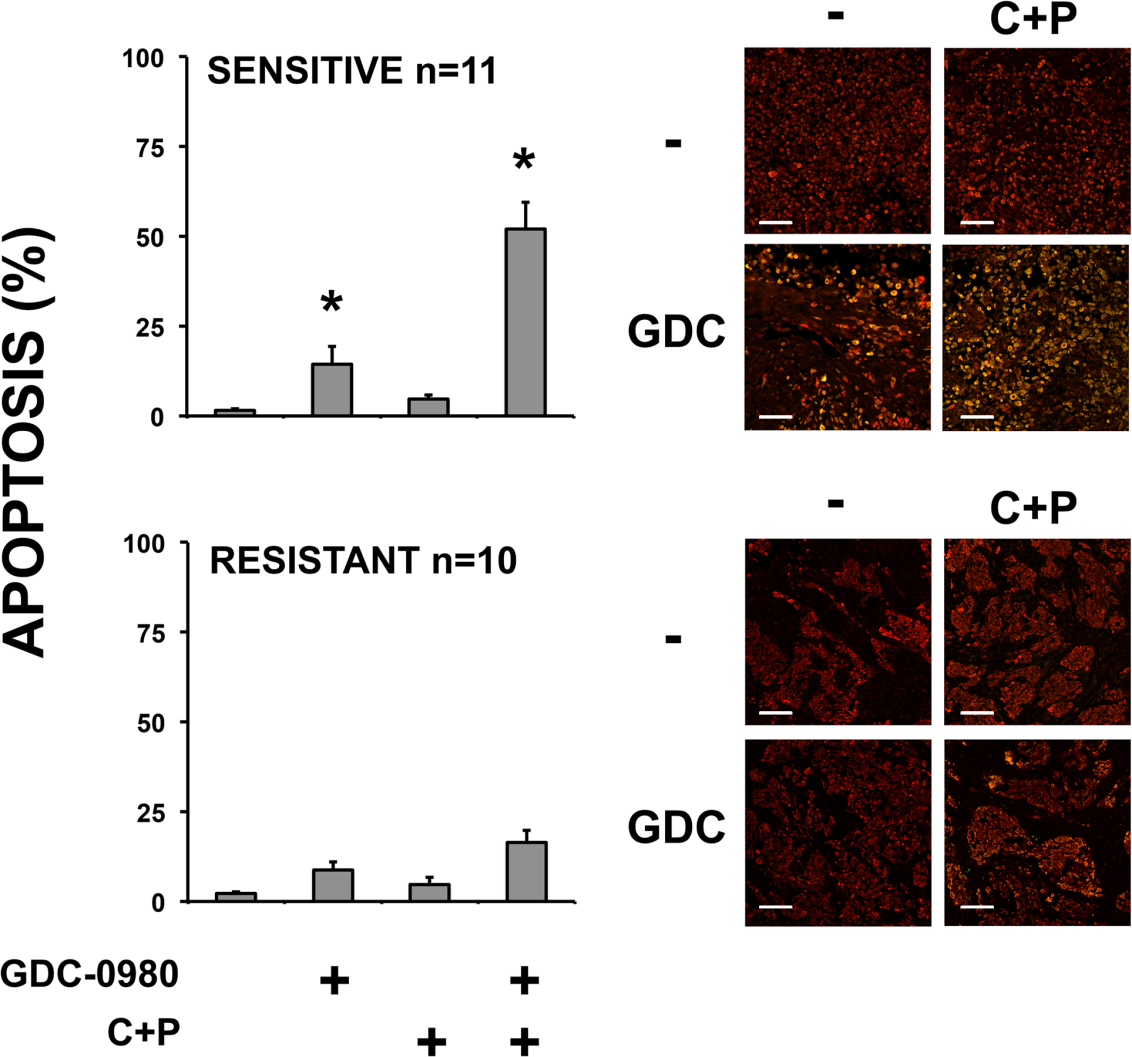
A subset of mesothelioma tumor fragment spheroids is sensitive to GDC-0980. Tumor fragments spheroids grown from 21 patient tumor samples were treated with GDC-0980 (1 μM), cisplatin (200 μM) plus pemetrexed (10 μM)(C+P) or the combination for 24 h. Cleaved caspase 3 (green) and pan-cytokeratin (red) immunofluorescence was detected and the merging of signals (*yellow*) indicates mesothelioma cells with caspase activation. One hundred cells were counted in triplicate from different microscopic fields. Eleven tumors showed a significant response to GDC-0980 (*sensitive*) while 10 were unresponsive (*resistant*) (*, *p* < *0*.*05* compared to the same treatment without GDC-0980; mean ± SEM). Images are representative of the tumor fragment spheroids in each group. (*Sensitive*, *top; resistant*, *bottom–scale bar 100*μ*m)*.

To test our findings in actual tumor from patients, we treated 21 tumor fragment spheroids with GDC-0980, cisplatin plus pemetrexed, and their combination. As found in multicellular spheroids, we identified a group of tumors sensitive to GDC-0980 (n = 11) and another group resistant (n = 10) (Fig 2 – additional images in S2 Fig).

### The response to GDC-0980 does not correlate with activation of the Akt/mTOR pathway in *ex vivo* tumor fragment spheroids

**Fig 3:**
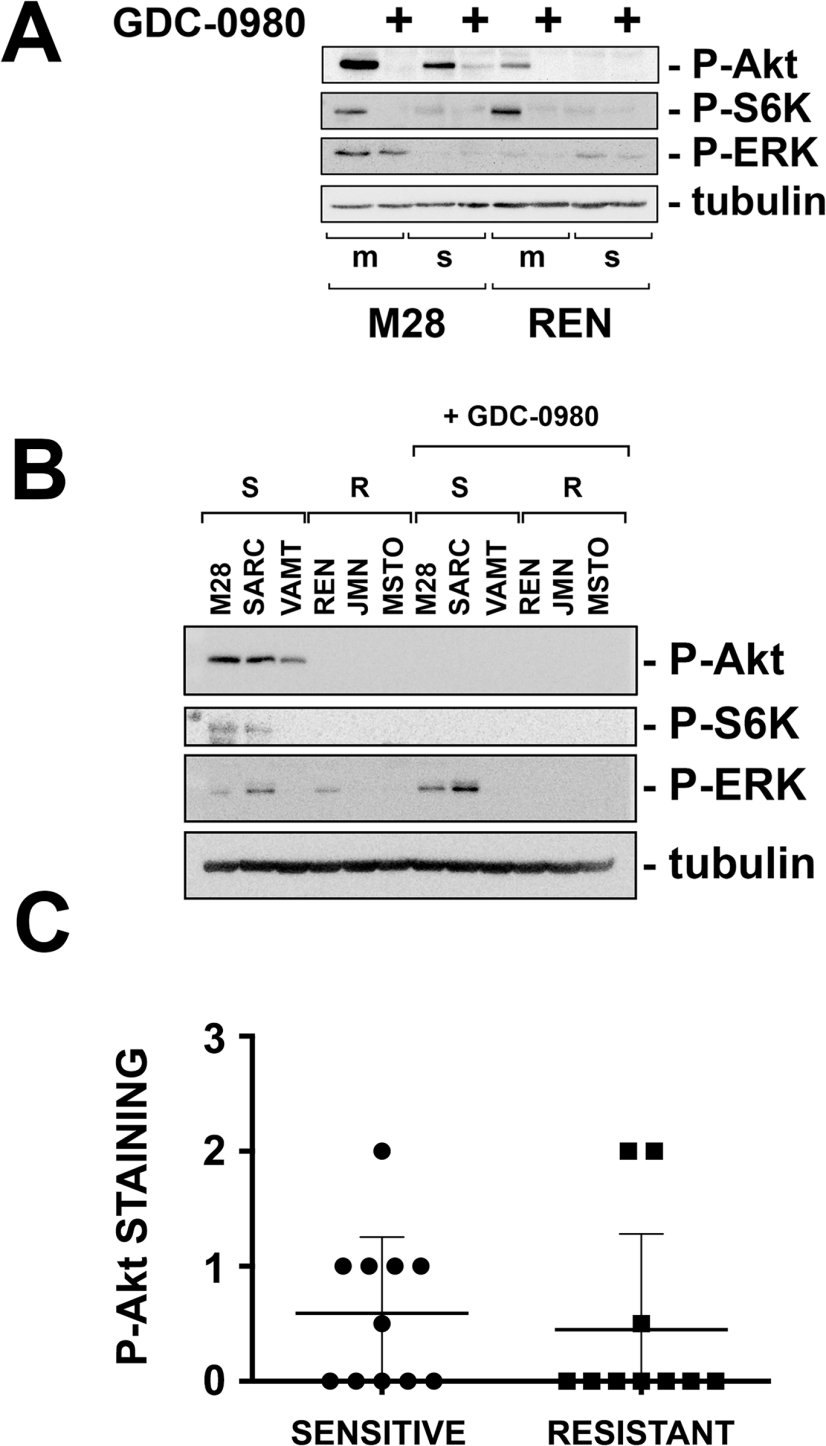
GDC-0980 efficacy does not correlate with Akt/mTOR activation in tumor fragment spheroids. (A) M28 and REN cells grown as monolayers (*m*) or spheroids (*s*) were treated with GDC-0980 (1 μM) for 6 h and studied by immunoblotting for phosphorylated Akt, S6K and ERK. Mesothelioma cells down-regulated the Akt/mTOR pathway when grown as 3D multicellular spheroids, as we have previously shown [1]. GDC-0980 inhibited P-Akt and P-S6K in both monolayers and spheroids. P-ERK, used as a control, was not affected by GDC-0980. (B) GDC-*sensitive* (*S*) and-*resistant* (*R*) spheroids were treated with GDC-0980 (1 μM) for 6 h and analyzed by immunoblotting for P-Akt, P-S6K and P-ERK. Multicellular spheroids sensitive to GDC-0980 had higher P-Akt than resistant ones. (**C**) The 21 tumor fragment spheroids with a known response to GDC-0980 (see Fig 2) were studied for expression of P-Akt by immunohistochemistry. In the tumor fragments, in contrast to the multicellular spheroids, response to GDC-0980 did not correlate with P-Akt levels *(p = 0*.*6713* sensitive vs resistant, n = 11 for the sensitive group, n = 10 for the resistant group).

GDC-0980 has been described as having robust activity in PI3K-driven cancers [15] and to be effective in mesotheliomas with highly active Akt [12]. We thus correlated the response to GDC-0980 with the activity of this pathway. First, we confirmed that GDC-0980 inhibited P-Akt in M28 and REN monolayers and spheroids (Fig 3A). Then, we studied the 6 cell lines as multicellular spheroids to compare their response to GDC-0980 with the levels of P-Akt ^Ser473^, P-S6K ^Thr389^ and, as a representative from another pathway, P-ERK. In the full panel of cell lines studied as spheroids, we found that GDC-0980 response appeared to correlate with higher baseline P-Akt ^Ser473^ and P-S6K ^Thr389^ levels (Fig 3B). Next, we wanted to know if the more complex tumor fragment spheroids would show a similar relationship between response to GDC-0980 and baseline activation of the PI3K/Akt pathway; hence, we measured P-Akt^Ser473^ levels in the 21 tumor fragment spheroids shown to be sensitive or resistant to GDC-0980 (see Fig 2). Surprisingly, in contrast to the findings in multicellular spheroids, in the tumor fragment spheroids, we found no correlation between the response to GDC-0980 and the staining intensity for P-Akt (Fig 3C). In addition, we also found no correlation between the response to GDC-0980 and several measures of the PI3K/mTOR pathway: P-S6K ^Thr389^ (a target of mTOR), GLUT-1 (a measure of Akt activity on glycolysis [31, 32]), Ki67 (a marker of cell proliferation), or IRS-1 (regulated by Akt and mTOR [33]) (S3 Fig). We also found no relationship between the response to GDC-0980 and the levels of the pro-apoptotic BH3-only protein, Bim (as a measure of the apoptotic priming displayed by the specimens [27]). Moreover, there was no correlation between the response to GDC-0980 and PET-derived indices of tumor glycolytic activity, such as total lesion glycolysis (TLG) and mean SUVmax, recently shown to correlate with survival in patients with mesothelioma (S3 Fig). [34]. Thus, the apoptotic response to GDC-0980 in the tumor fragment spheroid model did not clearly relate to the activity of the PI3K/mTOR pathway.

### GDC-0980 does not require Bim or Bid in the apoptotic cascade

**Fig 4:**
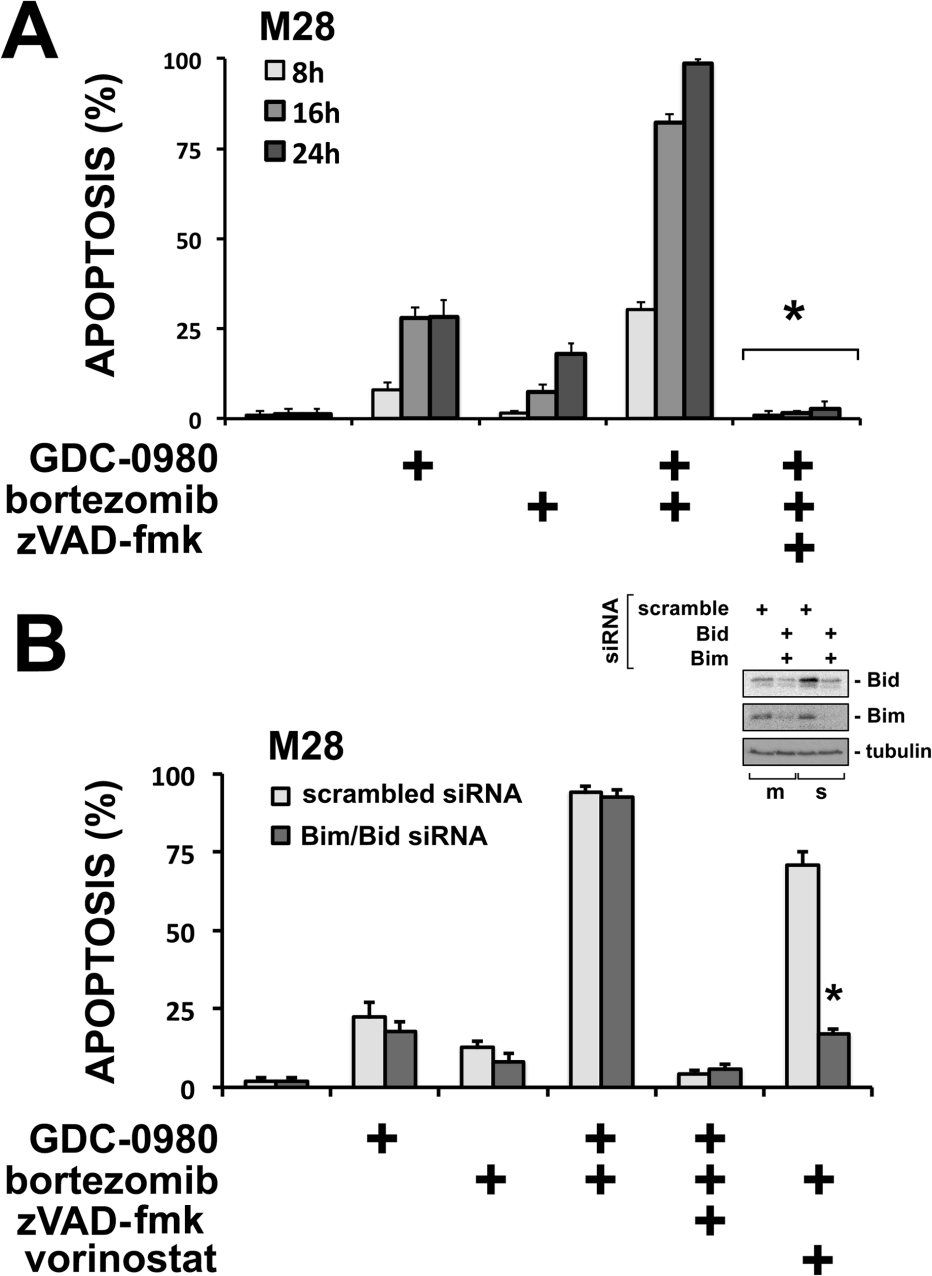
GDC-0980 efficacy is caspase-dependent but does not require Bim or Bid. (A) M28 spheroids were treated with GDC-0980 (1 μM), bortezomib (25 nM) or the combination with or without the pan-caspase inhibitor zVAD-fmk (20 μM) for 8, 16 and 24 h. Apoptosis was measured by counting nuclear condensation of disaggregated cells stained with Hoechst. GDC-0980 activity was completely abolished by zVAD-fmk at all time points. (* p < *0*.*05* different from GDC-0980 plus bortezomib; *n* = 3; mean ± SD). (B) M28 spheroids, transfected with a control scrambled siRNA (10 nM) or with a combination of Bim siRNA and Bid siRNA (10 nM), were treated with GDC-0980 (1 μM), bortezomib (25 nM), or the combination with or without zVAD-fmk and apoptosis measured. SAHA (5 μM) was used as positive control for a requirement for Bim/Bid, as we have previously shown [28]. Efficacy of knockdown was measured by immunoblot (see insert). Response to GDC-0980 was not affected by Bim/Bid siRNA but was abolished by zVAD-fmk. The response to SAHA was significantly reduced by the Bim/Bid knockdown, confirming effective knockdown. (* p < *0*.*05* different from scrambled control siRNA; *n* = 3; mean ± SD). In sum, the apoptotic response to GDC-0980 does not require Bim and Bid [1, 27, 28, 55].

To confirm that the effects of GDC-0980 are mainly routed via apoptosis, we blocked the activation of caspases with zVAD-fmk, a pan-caspase inhibitor. Indeed, the response to GDC-0980, bortezomib or the combination was completely blocked by zVAD-fmk as early as 8 h of treatment (Fig 4A–additional data in S4 Fig), a result confirmed also by another group in pancreatic cancer [19]. However, the response to GDC-0980 was not affected by the ablation of Bim and Bid, key BH3-only proteins of the intrinsic and extrinsic apoptotic pathways respectively, which we previously found to be required for the apoptotic response of mesothelioma [1, 25, 27, 28](Fig 4B). This suggests that GDC-0980 induces apoptosis without the requirement for Bim or Bid.

One cellular program that can activate caspases without the involvement of the intrinsic and extrinsic apoptotic pathways is autophagy. Designed to maintain cell survival under stress, autophagy relies on the lysosomal degradation pathway to recycle cellular material to provide sufficient nutrients for the cells (termed *productive autophagy*). However, beyond a certain threshold, autophagy can kill stressed cells by activating caspases and exploiting components of the apoptotic machinery [35] (termed *abortive autophagy*). We then asked whether autophagy was active in our system and, if so, whether autophagy affected the response to GDC-0980.

### GDC-0980 activates autophagy in the sensitive spheroids only

Autophagy is a multi-step dynamic process by which cellular material is engulfed in autophagic vesicles, delivered to the lysosomes, and degraded. To measure autophagic flux, one must inhibit the lysosomal proteases and detect the accumulation of autophagic proteins. One protein commonly measured is LC3-II, a conjugate form of LC3-I, which is recruited to the autophagosomes [36]. Thus, we treated our panel of multicellular spheroids with GDC-0980 in the presence or absence of ammonium chloride (NH_4_^+^) which inhibits late stage autophagy by blocking the acidification of lysosomes, thereby inhibiting the lysosomal proteases and causing the accumulation of undigested materials and LC3-II in the vesicles.

**Fig 5:**
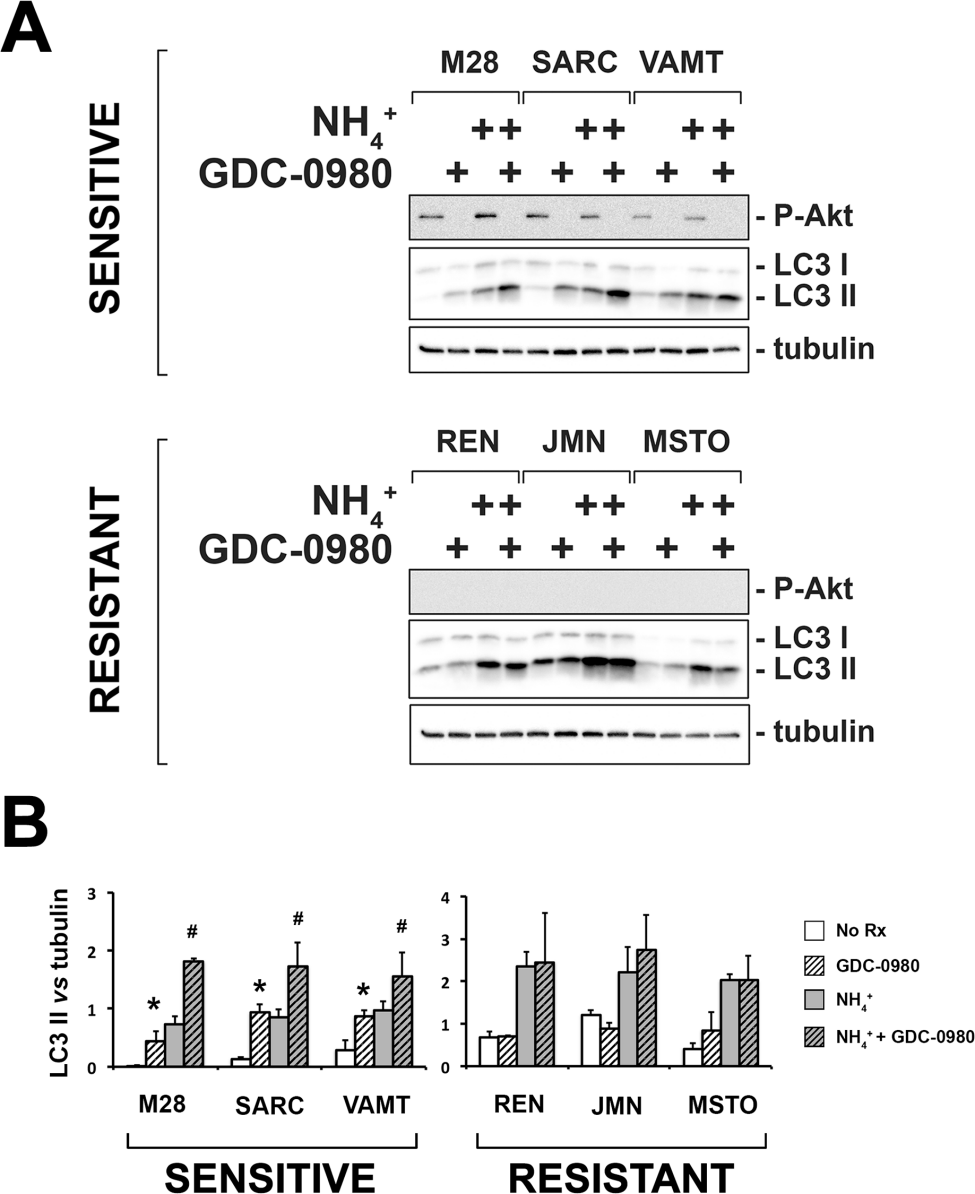
GDC-0980 induces autophagy only in the sensitive spheroids. (A) Multicellular spheroids *sensitive* (M28, SARC, VAMT) and *resistant* (REN, JMN, MSTO-211H) to GDC-0980 were treated with GDC-0980 (1 μM), ammonium chloride (NH_4_^+^, 10 mM) or the combination for 6 h. LC3 levels were assessed by immunoblot. P-Akt levels were measured to confirm GDC-0980 activity. GDC-0980 increased LC3-II levels only in the *sensitive* spheroids; this was shown at baseline and also after blockade of autophagy with ammonium chloride. On the contrary, GDC-0980 did not increase LC3 II protein levels in the resistant cell lines, with or without ammonium chloride. (B) The band intensities in 3 separate immunoblots were measured by densitometry and the ratios of LC3 II to tubulin values (LC3 vs tubulin) are shown. Statistical significance was calculated by ANOVA with Tukey’s test (* untreated vs GDC-0980, **#** NH_4_^+^ vs NH_4_^+^+GDC-0980; *p < 0*.*05*; n = 3; mean ± SD).

We found that spheroids from all six cell lines had autophagic flux at baseline, as determined by an increase in the amount of LC3-II after blockade with ammonium chloride (Fig 5), a result we confirmed with the detection of LC3 puncta by immunofluorescence (S5 Fig). GDC-0980 increased autophagy further, but only in the sensitive spheroids (Fig 5). In tumor fragment spheroids also, GDC-0980 increased autophagy, as shown by the formation of LC3 puncta, only in the sensitive group (S6 Fig). Altogether these results suggest that autophagy is responsive to PI3K/Akt blockade only in the spheroids sensitive to GDC-0980 and that its induction might be involved in the response to GDC-0980. We then determined whether the activity of GDC-0980 correlated with differences in autophagy in the two groups of mesotheliomas.

### ATG13 is a marker of sensitivity to GDC-0980

The activation of the ATG13/ULK complex is considered one of the major regulatory inputs that controls the autophagic process and is the step most closely associated with mTOR regulation [37, 38]. The mTOR substrate complex (ATg13/ULK1/FIP200/ATG101) [39] acts as a node for integrating incoming autophagy signals into formation of autophagosomes, a step that involves the phosphorylation of ATG13 and the accumulation of ATG13 into aggregates called puncta at assembly sites [38, 40–45]. Hence, we imaged spheroids grown from the six cell lines to detect cells exhibiting ATG13 puncta.

**Fig 6:**
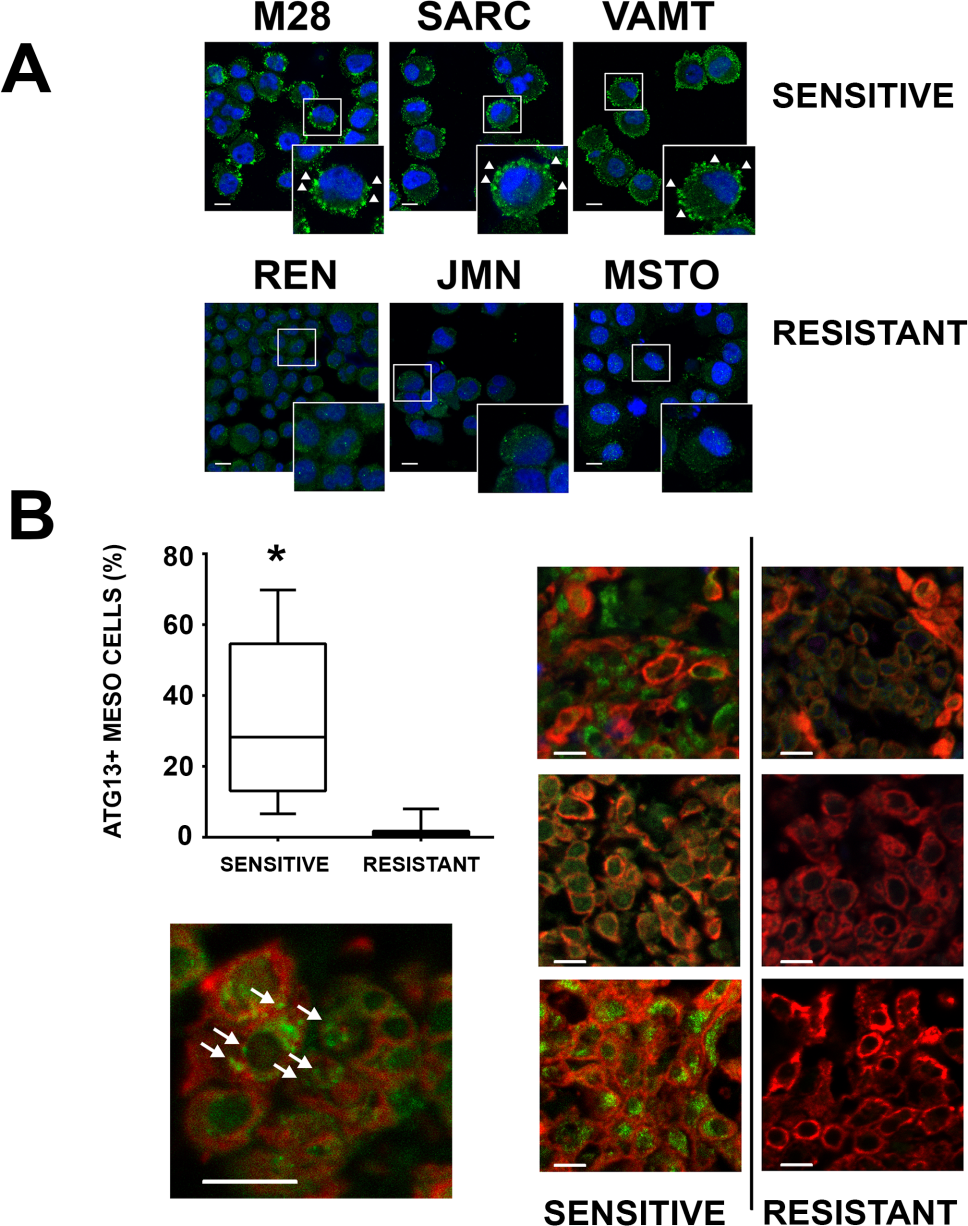
ATG13 puncta are present only in the spheroids sensitive to GDC-0980. (A) Multicellular spheroids found to be *sensitive* (M28, SARC, VAMT) or *resistant* (REN, JMN, MSTO-211H) to GDC-0980 were immunostained for ATG13. Only the spheroids sensitive to GDC-0980 displayed ATG13 puncta. (B) Tumor fragment spheroids that were found to be sensitive or resistant to GDC-0980 were stained for ATG13. All the tumor fragment spheroids sensitive to GDC-0980 had more than 10% cells positive for ATG13 puncta, whereas the resistant group showed little to no ATG13 staining. (* p < *0*.*05* sensitive vs resistant; n = 11 for the sensitive group, n = 10 for the resistant group; mean ± SEM). Panels are representative images of tumor fragment spheroids stained for pan-cytokeratin (*red*) and ATG13 (*green*). An enlarged panel is also provided showing ATG13 puncta in a sensitive tumor fragment spheroid, as indicated by arrows (*scale bar for both panels 10*μ*m*).

We found constitutive ATG13 puncta only in the multicellular spheroids sensitive to GDC-0980 (Fig 6A). To confirm these findings, we then assessed the presence of ATG13 puncta in tumor fragment spheroids. Again, we found that mesothelioma cells with ATG13 puncta were commonly seen in the spheroids of the sensitive group and rarely seen in the spheroids from the resistant group (Fig 6B). Response to GDC-0980 (measured as the additional apoptosis induced when GDC-0980 was added to cisplatin plus pemetrexed) showed a linear correlation with the percentage of cells with ATG13 puncta (R = 0.72) (S7 Fig). Thus, autophagy, as measured by the presence of ATG13 puncta, correlated with the responsiveness to GDC-0980 in both 3D models of mesothelioma.

### Inhibition of autophagy in the M28 spheroids increases their response to chemotherapy

**Fig 7:**
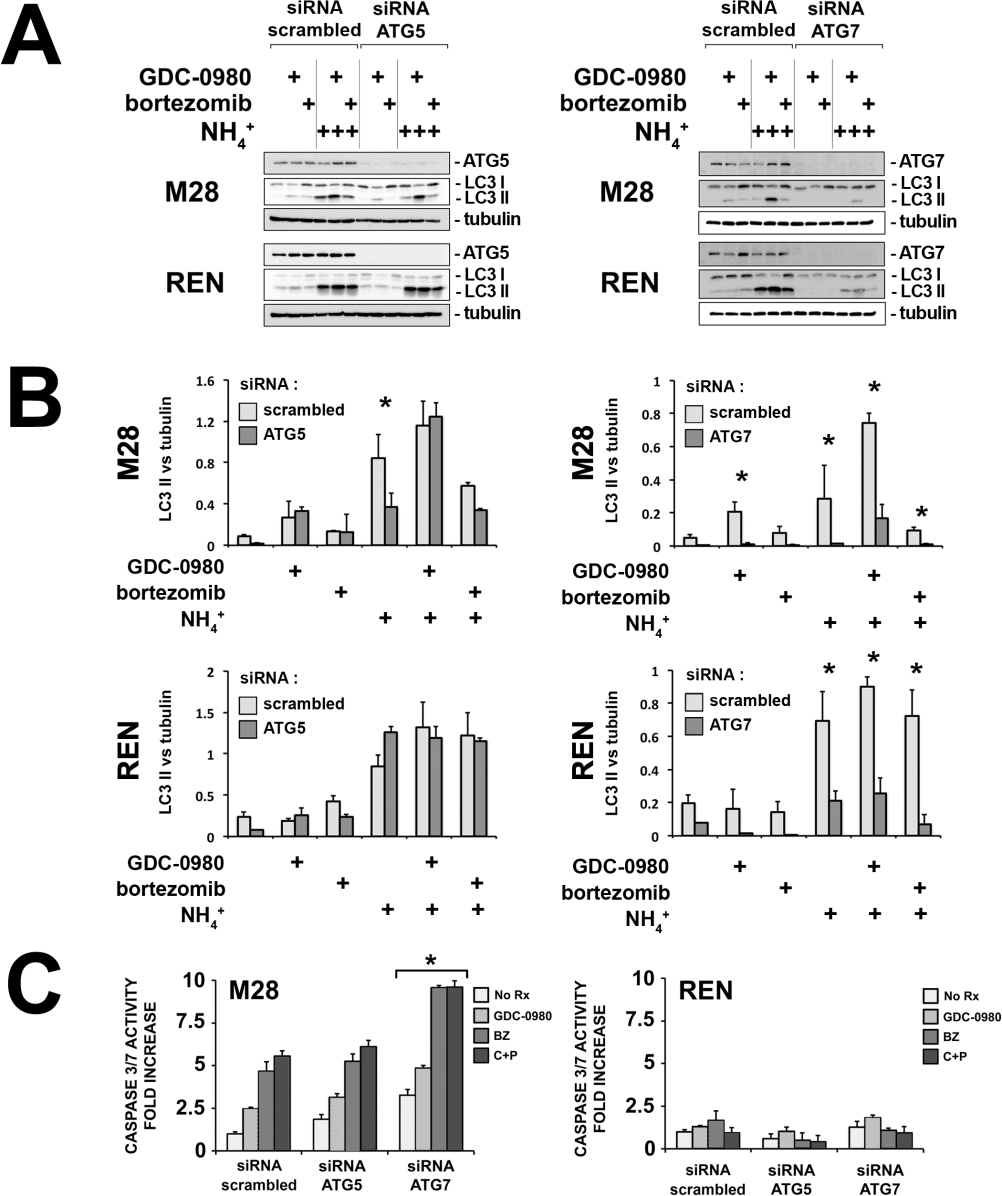
Inhibition of autophagy potentiates the response to chemotherapy only in the spheroids sensitive to GDC-0980. (A) M28 and REN spheroids were transfected with ATG5 (left panels) and ATG7 siRNA (right panels) and treated with GDC-0980 (1 μM), bortezomib (25 nM), ammonium chloride (NH_4_^+^, 10 mM) or the combination for 6 h. Both ATG5 and ATG7 siRNA effectively reduced their respective protein levels to less than 10% of the original levels. However, in M28 and REN spheroids, ATG5 siRNA failed to reduce LC3-II levels, showing that autophagy was not inhibited. Instead, in both M28 and REN spheroids, ATG7 siRNA decreased LC3-II levels, showing that it was effective in blocking autophagy. Of note, bortezomib did not affect the autophagic flux of either spheroid. (B) The band intensities in 3 separate immunoblots were measured by densitometry and the ratios of LC3 II to tubulin values (LC3 vs tubulin) are shown. Statistical significance was calculated by ANOVA with Tukey’s test (* scrambled siRNA vs ATG5 or 7 siRNA; *p < 0*.*05*; n = 3; mean ± SD). (C) M28 and REN spheroids, grown from cells transfected with ATG5 or ATG7 siRNA, were treated with GDC-0980 (1 μM), bortezomib (25 nM)(BZ) or cisplatin (200 μM) plus pemetrexed (10 μM)(C+P) for 16 h. Apoptosis was measured by a CaspaseGlo assay. The response to GDC-0980 and to chemotherapy was potentiated by ATG7 siRNA, only in M28 spheroids. (* p < *0*.*05* compared to scrambled control siRNA; *n* = 3; mean ± SD).

To investigate the role of autophagy, we evaluated the effects of the ablation by siRNA of two essential components of the autophagic machinery, ATG5 or ATG7, in M28 and REN spheroids. Using ATG5 siRNA, we were not able to block the autophagic flux in both M28 and REN spheroids completely, as shown by the continued accumulation of LC3-II after ammonium chloride (Fig 7A and 7B, left panels). Indeed, it is known that small amounts of ATG5 can still support autophagy [46]. Instead, ATG7 siRNA was more effective in blocking autophagy (Fig 7A and 7B, right panels). Accordingly, whereas ATG5 siRNA had little effects on the survival of M28 and REN spheroids, ATG7 siRNA induced a significant amount of cell death in the absence of treatments and further increased the responsiveness to GDC-0980 and to chemotherapy, an increase in chemosensitivity seen only in M28 spheroids (Fig 7C). These results provide evidence for a survival role for autophagy in mesothelioma 3D models [47, 48].

## Discussion

Cancers are now known to be heterogeneous in their strategies for survival [49]. Whereas such heterogeneity limits the use of any single targeted approach, it also provides opportunities to identify responsive subsets of tumors. In this study, we have identified subsets of mesothelioma that are sensitive to GDC-0980, a novel dual PI3K/mTOR inhibitor currently in clinical trials. Interestingly, this sensitivity did not correlate as expected with the phosphorylation of Akt and S6K [15], downstream targets of PI3K and mTOR activation. Instead, we found a correlation with autophagy, a pathway providing nutritional support during times of cell stress. The presence of ATG13 puncta, a feature of early autophagy regulation by the mTOR substrate complex, may identify tumors that are susceptible to the action of GDC-0980.

Whereas other studies have examined autophagy in mesothelioma [48, 50], this is the first to focus on the use of 3D models. In contrast to standard bi-dimensional (2D) monolayers, a 3D setting is similar to the “least avascular unit”, the unit of tumor that lies between capillaries and is most limited in its nutrient supply [51]. Hence, the 3D setting promises to be more appropriate for the study of the role of autophagy in tumors. This is also the first study to utilize *ex vivo* tumor tissue to measure autophagy. In fact, using tumor fragment spheroids derived from patient tumors, we confirmed our findings from the cell lines, showing that the response to GDC-0980 correlates with the presence of ATG13 puncta.

By using these 3D models, we have found that there is a group of tumors with autophagy that is sensitive to PI3K/mTOR inhibition with GDC-0980. We suspect that these tumors are under stress and using autophagy, at least in part, as a survival mechanism. After GDC-0980, both autophagy and cell death increase; either the autophagy is counteracting the stress and acting in a survival capacity or is itself inducing death. With the inhibition of autophagy, the survival role of the autophagy becomes more clear because, without autophagy, the cells become more susceptible to stresses such as chemotherapy. The complexity concerning the role(s) of autophagy may be in concert with the proposed context-dependent, dual role for autophagy in cancer: *productive*, with survival roles whose inhibition leads to cell death, or *abortive*, leading to cell death when excessively or inappropriately induced [47]. On the other hand, in the other group of tumors that are resistant to GDC-0980, autophagy does not respond to PI3K/mTOR inhibition and the inhibition of autophagy has no effect on cell survival. We do not yet know whether the lack of an increase in autophagy in the resistant tumors indicates that they are not being stressed or because autophagy is defective: these scenarios have been described in other tumors [52, 53]. It is also possible that autophagy in the resistant group is not regulated by the mTOR substrate complex.

In our tumor samples, the phosphorylation status of Akt did not correlate with the activity of the PI3K/mTOR inhibitor [1, 2, 54]. It may be that, in the tumor, a more complex system than that of cell lines, the phosphorylation of Akt did not fully represent the activity of the pathway or that the effectiveness of inhibition did not depend on the level of activity of the pathway. Interestingly, despite the lack of correlation with the phosphorylation of Akt/mTOR, the response to GDC-0980 did correlate with autophagy, as measured by the presence of ATG13 puncta. To our knowledge, this is the first time that sensitivity to GDC-0980 has been shown to be associated with autophagic activity, either in cell lines or importantly in tumor samples studied *ex vivo*.

Before attempting to target autophagy therapeutically, one would want to identify tumors responsive to this approach, currently a difficult proposition because of the inability to measure autophagy confidently in fixed or *in vivo* tumor tissue [46]. We have found that the presence of ATG13 puncta appear to identify these tumors, at least in mesothelioma. Our preliminary studies suggest that ATG13 may not be required to drive autophagy, as shown elsewhere [41], but may serve as a marker of tumors sensitive to PI3K/mTOR inhibition. Conversely, the resistant group showed no ATG13 puncta and demonstrated a basal autophagy that did not respond to PI3K/mTOR inhibition and whose inhibition had no effect on the cells. Such tumors would presumably be unresponsive to efforts to manipulate autophagy but could perhaps be targeted by other approaches.

## Conclusions

In this study, we have shown for the first time that GDC-0980 is active in a subset of mesotheliomas, which can be identified by the presence of ATG13 puncta. By studying 3D models of mesothelioma, we have found that, whereas all spheroids demonstrated basal autophagy, only the sensitive spheroids displayed ATG13 puncta at baseline, increased autophagy in response to GDC-0980 and became more chemoresponsive with inhibition of autophagy. Hence, autophagy represents a possible therapeutic target in a subset of mesotheliomas and we propose the presence of ATG13 puncta as a promising autophagy marker to identify these tumors.

## Supporting Information

S1 Fig Normal mesothelial cells are resistant to GDC-0980. Multicellular spheroids grown from normal mesothelial cells (NMC) and M28 cells were treated with GDC-0980 (1 μM), bortezomib (25 nM) or the combination for 24 h. Spheroids were then disaggregated, fixed, stained with Hoechst and examined for apoptotic nuclear condensation. Neither GDC-0980 nor bortezomib had an effect in normal mesothelial cells when given alone or in combination. M28 spheroids were used as positive control for response to GDC-0980. (* *p* < 0.05, different from the same treatment without GDC-0980; n = 3; mean ± SD)
(TIF)

S2 Fig A subset of mesothelioma tumor fragment spheroids is sensitive to GDC-0980. (A) Immunofluorescent staining of tumor fragment spheroids treated with GDC-0980 (1 μM), cisplatin (200 μM) plus pemetrexed (10 μM)(C+P) or the combination for 24 h. Cleaved caspase 3 (green) and pan-cytokeratin (red) were detected; the merging of signals (*yellow*) indicates mesothelioma cells with caspase activation. (*scale bar 100*μ*m*) (B) Representative image of two tumor fragment spheroids after two weeks of cell culture. (*scale bar 500*μ*m*)
(TIF)

S3 Fig Response to GDC-0980 does not correlate with markers of the activation of the Akt/mTOR pathway. A tissue microarray comprising the original tumor from which tumor fragment spheroids were grown was analyzed by immunohistochemistry for P-S6K ^Thr389^, a downstream target of mTOR, Bim, a pro-apoptotic BH3-only protein, Ki67, a cell proliferation marker, and GLUT-1 and IRS-1, two markers of glucose metabolism connected with the Akt/mTOR pathway. Intensity staining for each protein was scored semi-quantitatively (0–4). The numbers over each bar represent first, the TLG (total lesion glycolysis) values calculated from the PET-CT scans for each patient, and second, the mean SUVmax values (see Methods). The response to GDC-0980 did not correlate with the stained proteins or with the TLG or mean SUVmax values.
(TIF)

S4 Fig GDC-0980 activity is completely inhibited by blocking caspase cleavage with zVAD-fmk. (A) Spheroids were grown from M28 cells and treated with GDC-0980 (1 μM), bortezomib (25nM) or the combination with or without zVAD-fmk (20 μM) for 24 h. Apoptosis was measured by Hoechst. zVAD-fmk completely blocked the apoptosis induced by either agent or the combination. (B) Cleaved caspase 3 was detected by immunoblot in M28 spheroids treated with GDC-0980 (1 μM), bortezomib (25nM) or the combination with or without zVAD-fmk (20 μM) for 16h. zVAD-fmk completely inhibited caspase cleavage due to either agent of the combination.
(TIF)

S5 Fig Both sensitive and resistant spheroids have basal autophagic flux. The spheroids were treated with 10 mM ammonium chloride (NH_4_^+^) for 8 h before harvesting. Spheroids were then trypsinized and cytospun on glass slides and stained for LC3 (green) and nuclei (TOPRO-3, blue). Magnified views of the regions in the dashed boxes are shown for representative cells with LC3 puncta (arrows). Both sensitive and resistant spheroids can be shown to display baseline autophagy because they accumulate LC3 puncta after exposure to NH_4_^+^. (*scale bar 10* μ*m*)
(TIF)

S6 Fig GDC-0980 induces autophagy only in the sensitive tumor fragment spheroids. (A) The 21 tumor fragment spheroids analyzed in Fig 2 were analyzed by immunofluorescence for the formation of LC3 puncta after GDC-0980 (1 μM–24 h). Representative images of the tumor fragment spheroids with or without GDC-0980 are shown (cytokeratin: red–LC3:green). (B) The percentage of mesothelioma cells positive for LC3 puncta was counted in triplicate for each tumor fragment spheroid. GDC-0980 significantly increased the number of cells with LC3 puncta only in the sensitive tumor fragment spheroids. (*p < *0*.*0001* GDC-0980 vs untreated control n = 11; mean ± SD). (*scale bar 50 μm*)
(TIF)

S7 Fig Response to GDC-0980 correlates with ATG13 puncta. 21 tumor fragment spheroids (resistant, circles; sensitive, triangles) were stained for ATG13 and cytokeratin. The response of the spheroids to GDC-0980 (displayed here as the difference between GDC-0980+C+P and C+P alone) was plotted against the percentage of mesothelioma cells (cytokeratin-positive) with ATG13 puncta; there was a linear correlation between the response to GDC-0980 and the presence of ATG13 puncta *(R = 0*.*72)*.
(TIF)

S1 Table Characteristics of tumors used to generate tumor fragment spheroids. Tumor fragment spheroids were grown from 11 epithelioid and 10 biphasic samples, with a range of tumor stages. Neither gender, histology nor stage was clearly associated with a response to GDC-0980.
(TIF)
