# Supplementary figures and images for: Autophagy Correlates with the Therapeutic Responsiveness of Malignant Pleural Mesothelioma in 3D Models
Source: PLoS One. 2015 Aug 18;10(8):e0134825. doi: 10.1371/journal.pone.0134825 (PMC4540424; doi:10.1371/journal.pone.0134825)

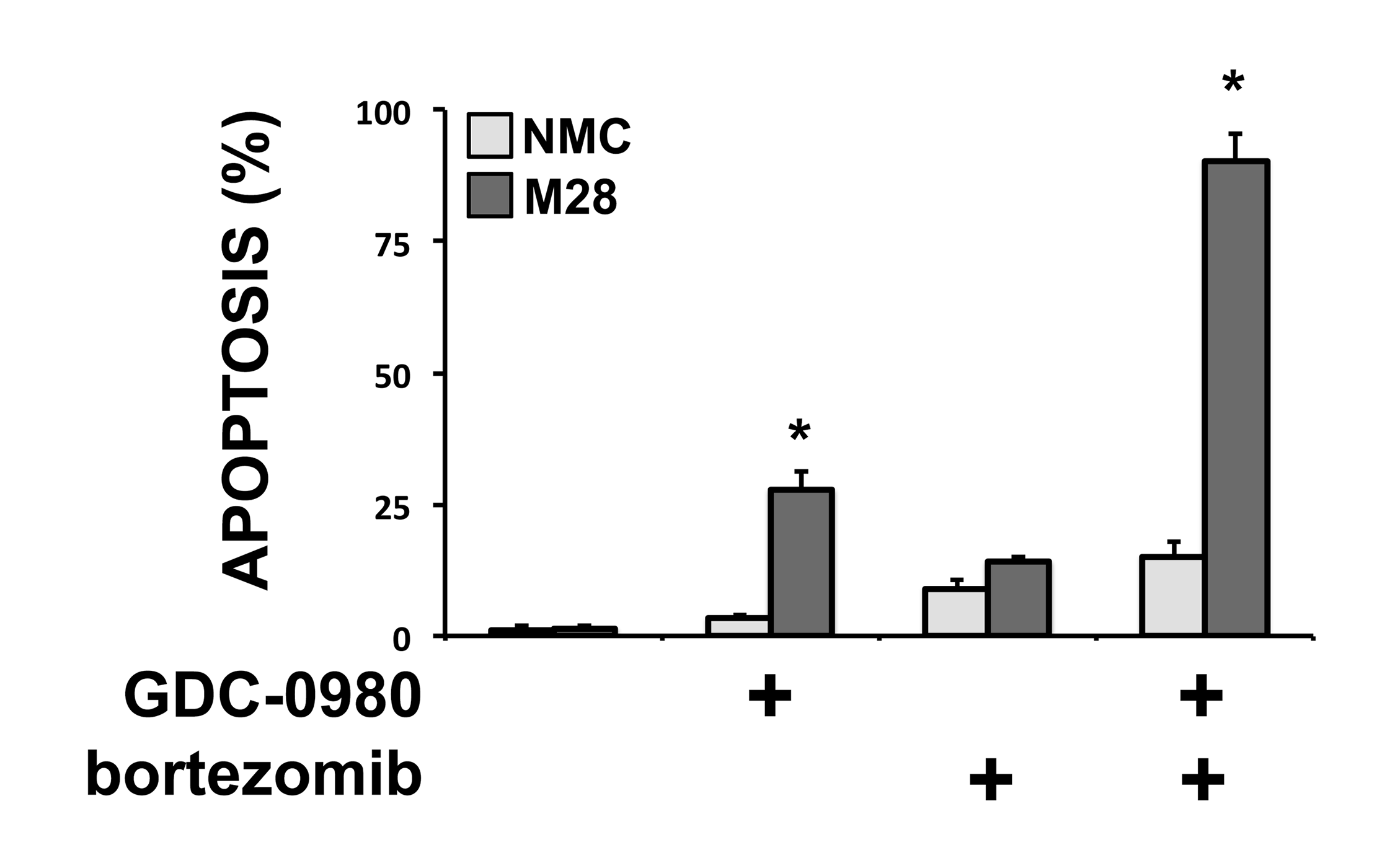

Supplement: S1 Fig — Multicellular spheroids grown from normal mesothelial cells (NMC) and M28 cells were treated with GDC-0980 (1 μM), bortezomib (25 nM) or the combination for 24 h. Spheroids were then disaggregated, fixed, stained with Hoechst and examined for apoptotic nuclear condensation. Neither GDC-0980 nor bortezomib had an effect in normal mesothelial cells when given alone or in combination. M28 spheroids were used as positive control for response to GDC-0980. (* p < 0.05, different from the same treatment without GDC-0980; n = 3; mean ± SD) (TIF) [file pone.0134825.s001.tif]

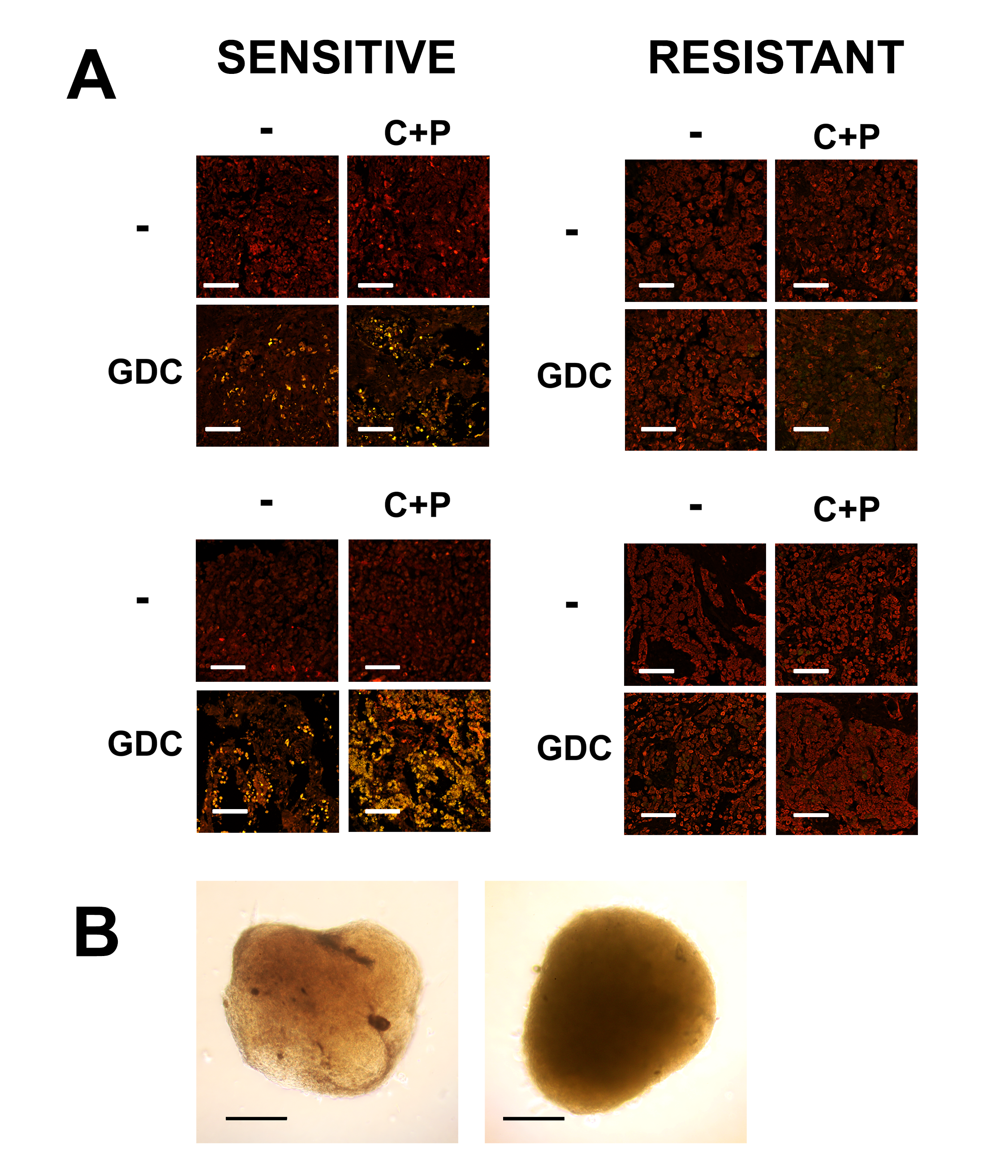

Supplement: S2 Fig — (A) Immunofluorescent staining of tumor fragment spheroids treated with GDC-0980 (1 μM), cisplatin (200 μM) plus pemetrexed (10 μM)(C+P) or the combination for 24 h. Cleaved caspase 3 (green) and pan-cytokeratin (red) were detected; the merging of signals (yellow) indicates mesothelioma cells with caspase activation. (scale bar 100μm) (B) Representative image of two tumor fragment spheroids after two weeks of cell culture. (scale bar 500μm) (TIF) [file pone.0134825.s002.tif]

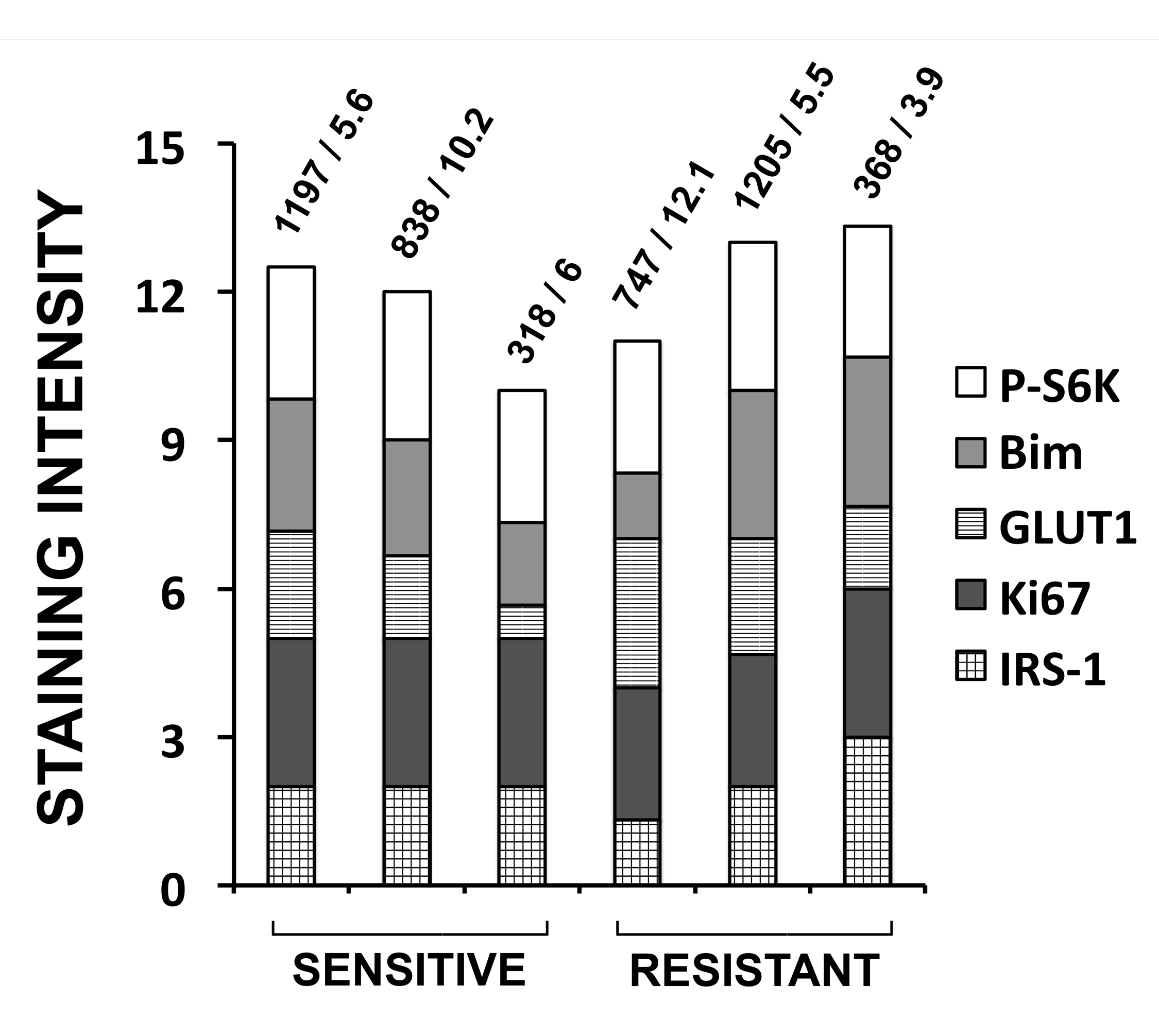

Supplement: S3 Fig — A tissue microarray comprising the original tumor from which tumor fragment spheroids were grown was analyzed by immunohistochemistry for P-S6K Thr389, a downstream target of mTOR, Bim, a pro-apoptotic BH3-only protein, Ki67, a cell proliferation marker, and GLUT-1 and IRS-1, two markers of glucose metabolism connected with the Akt/mTOR pathway. Intensity staining for each protein was scored semi-quantitatively (0–4). The numbers over each bar represent first, the TLG (total lesion glycolysis) values calculated from the PET-CT scans for each patient, and second, the mean SUVmax values (see Methods). The response to GDC-0980 did not correlate with the stained proteins or with the TLG or mean SUVmax values. (TIF) [file pone.0134825.s003.tif]

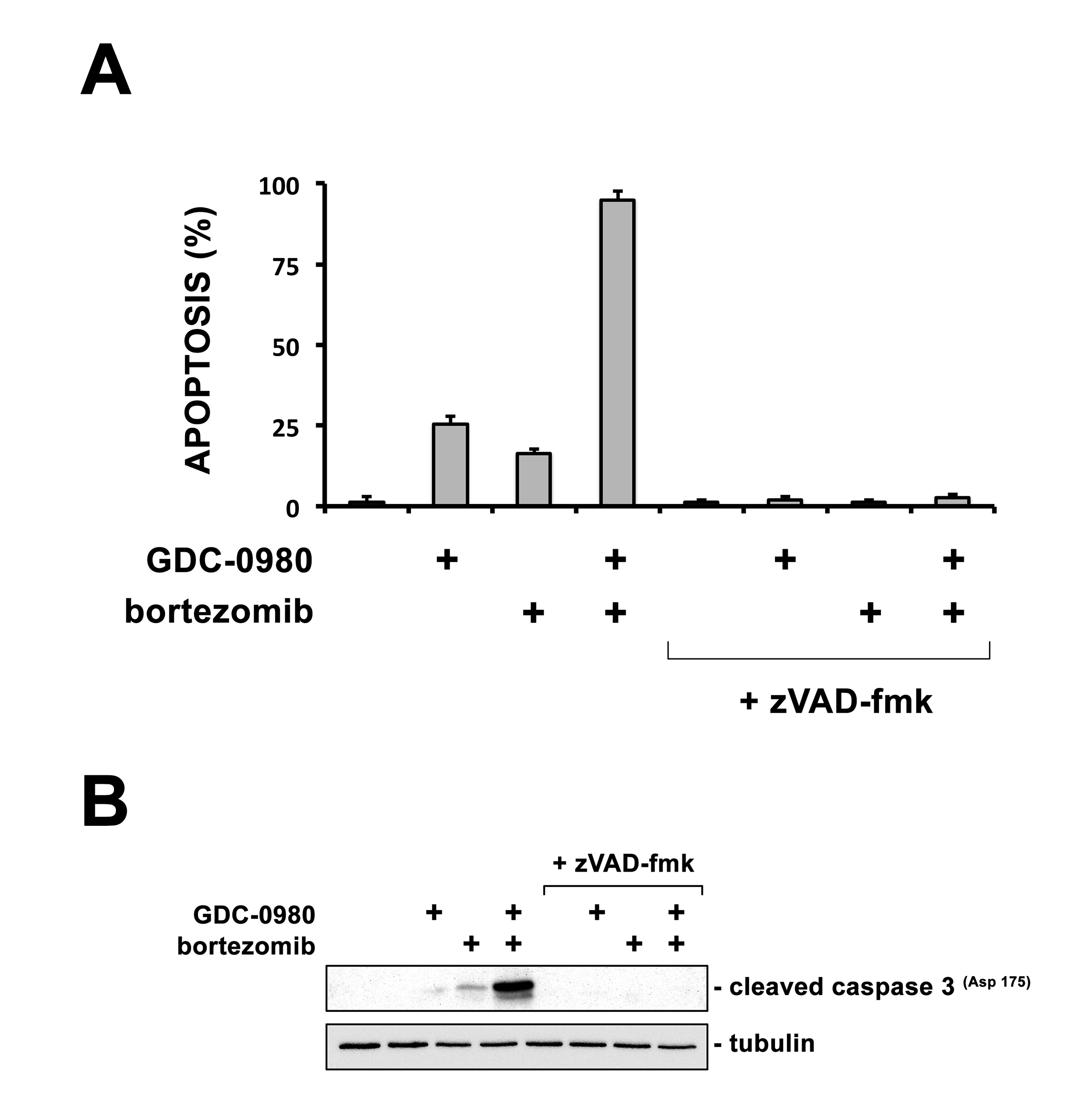

Supplement: S4 Fig — (A) Spheroids were grown from M28 cells and treated with GDC-0980 (1 μM), bortezomib (25nM) or the combination with or without zVAD-fmk (20 μM) for 24 h. Apoptosis was measured by Hoechst. zVAD-fmk completely blocked the apoptosis induced by either agent or the combination. (B) Cleaved caspase 3 was detected by immunoblot in M28 spheroids treated with GDC-0980 (1 μM), bortezomib (25nM) or the combination with or without zVAD-fmk (20 μM) for 16h. zVAD-fmk completely inhibited caspase cleavage due to either agent of the combination. (TIF) [file pone.0134825.s004.tif]

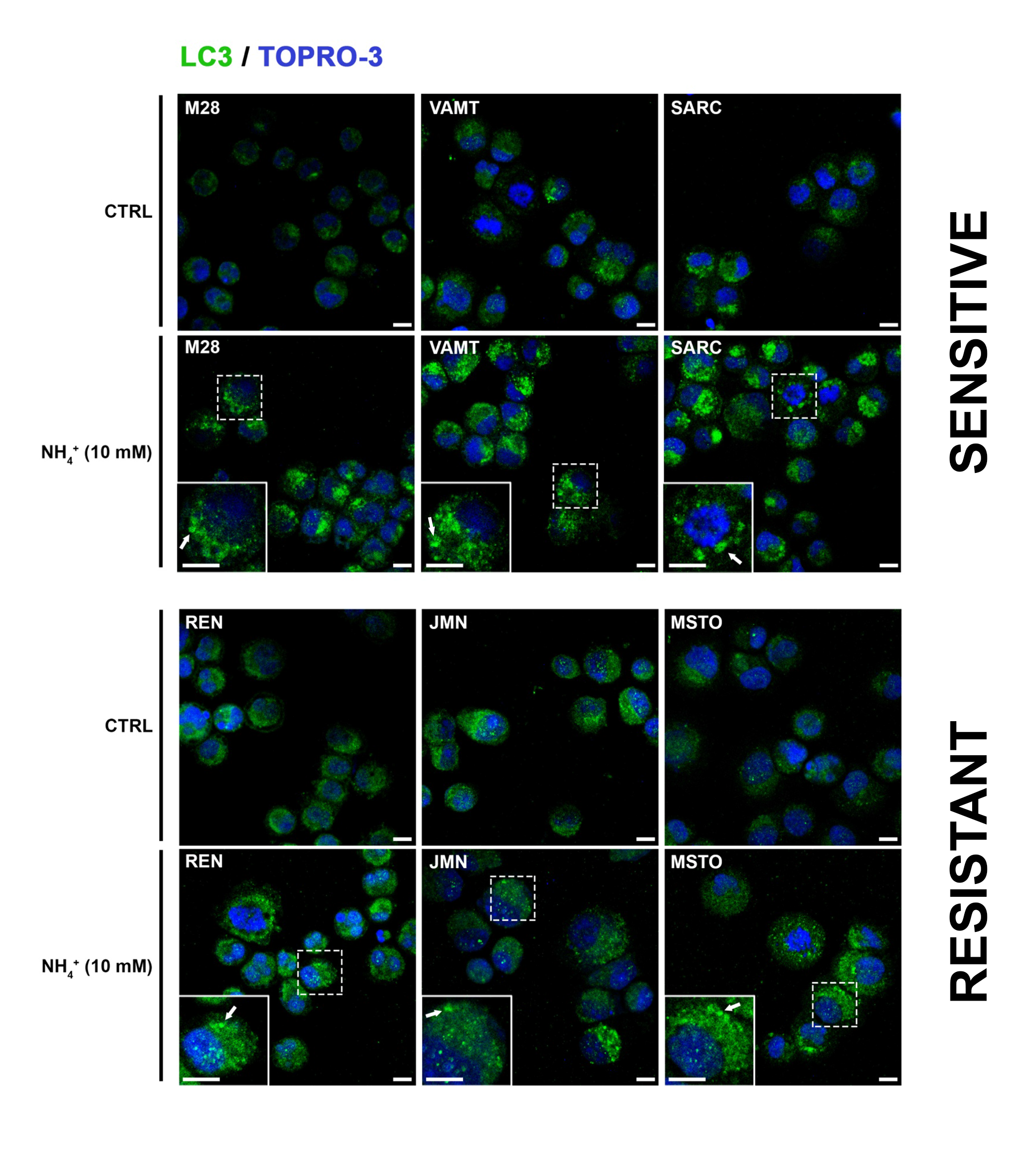

Supplement: S5 Fig — The spheroids were treated with 10 mM ammonium chloride (NH4 +) for 8 h before harvesting. Spheroids were then trypsinized and cytospun on glass slides and stained for LC3 (green) and nuclei (TOPRO-3, blue). Magnified views of the regions in the dashed boxes are shown for representative cells with LC3 puncta (arrows). Both sensitive and resistant spheroids can be shown to display baseline autophagy because they accumulate LC3 puncta after exposure to NH4 +. (scale bar 10 μm) (TIF) [file pone.0134825.s005.tif]

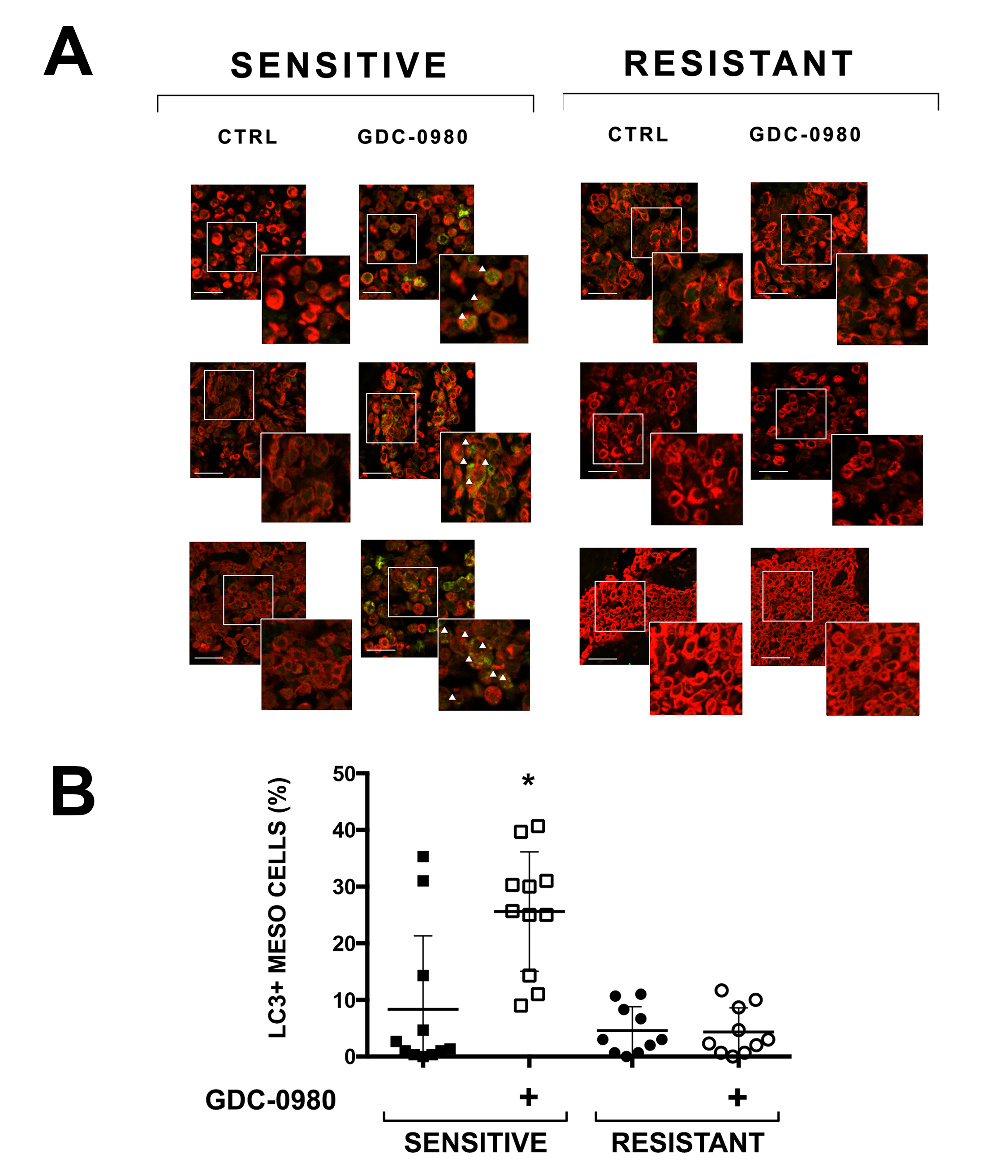

Supplement: S6 Fig — (A) The 21 tumor fragment spheroids analyzed in Fig 2 were analyzed by immunofluorescence for the formation of LC3 puncta after GDC-0980 (1 μM–24 h). Representative images of the tumor fragment spheroids with or without GDC-0980 are shown (cytokeratin: red–LC3:green). (B) The percentage of mesothelioma cells positive for LC3 puncta was counted in triplicate for each tumor fragment spheroid. GDC-0980 significantly increased the number of cells with LC3 puncta only in the sensitive tumor fragment spheroids. (*p < 0.0001 GDC-0980 vs untreated control n = 11; mean ± SD). (scale bar 50 μm) (TIF) [file pone.0134825.s006.tif]

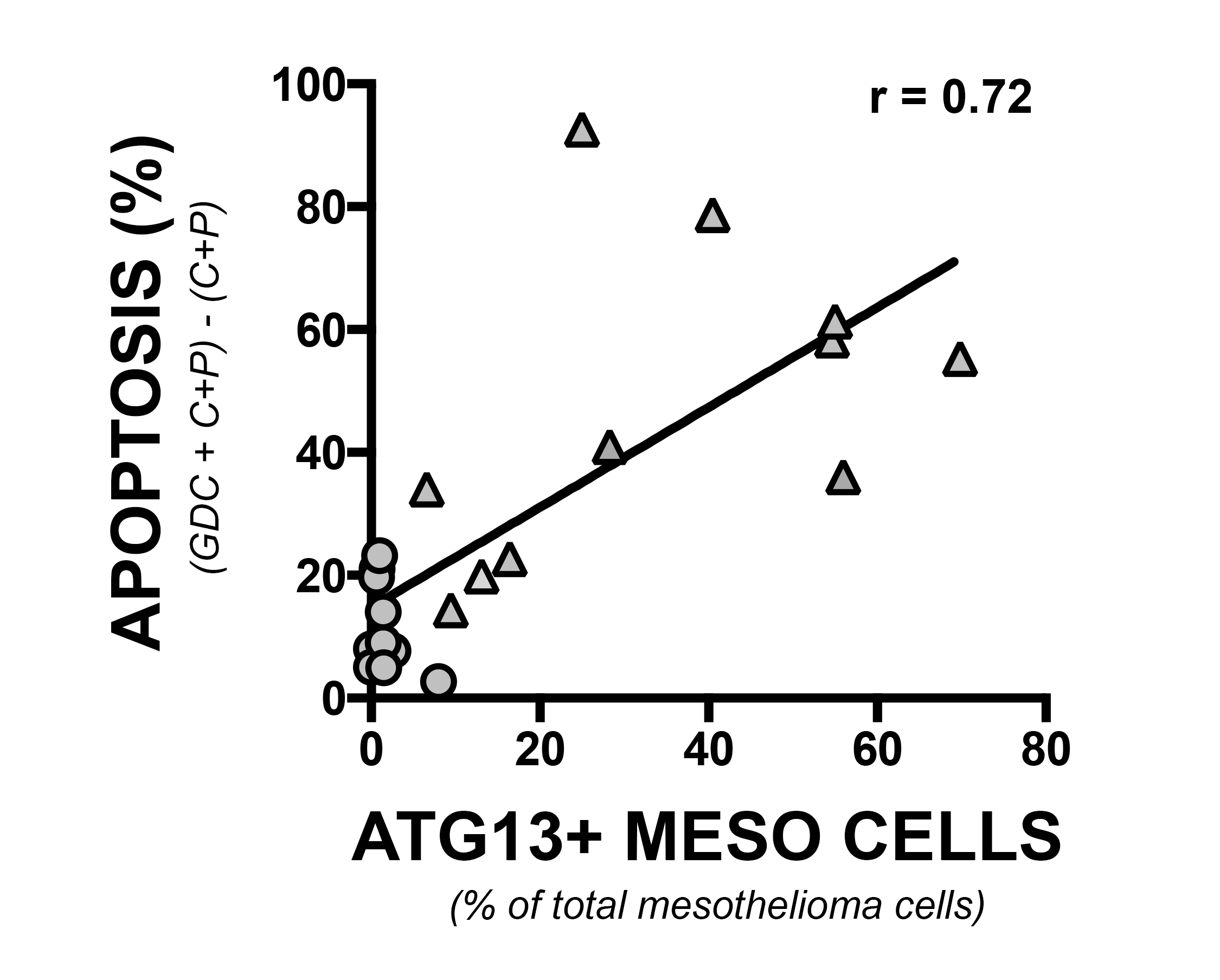

Supplement: S7 Fig — 21 tumor fragment spheroids (resistant, circles; sensitive, triangles) were stained for ATG13 and cytokeratin. The response of the spheroids to GDC-0980 (displayed here as the difference between GDC-0980+C+P and C+P alone) was plotted against the percentage of mesothelioma cells (cytokeratin-positive) with ATG13 puncta; there was a linear correlation between the response to GDC-0980 and the presence of ATG13 puncta (R = 0.72). (TIF) [file pone.0134825.s007.tif]

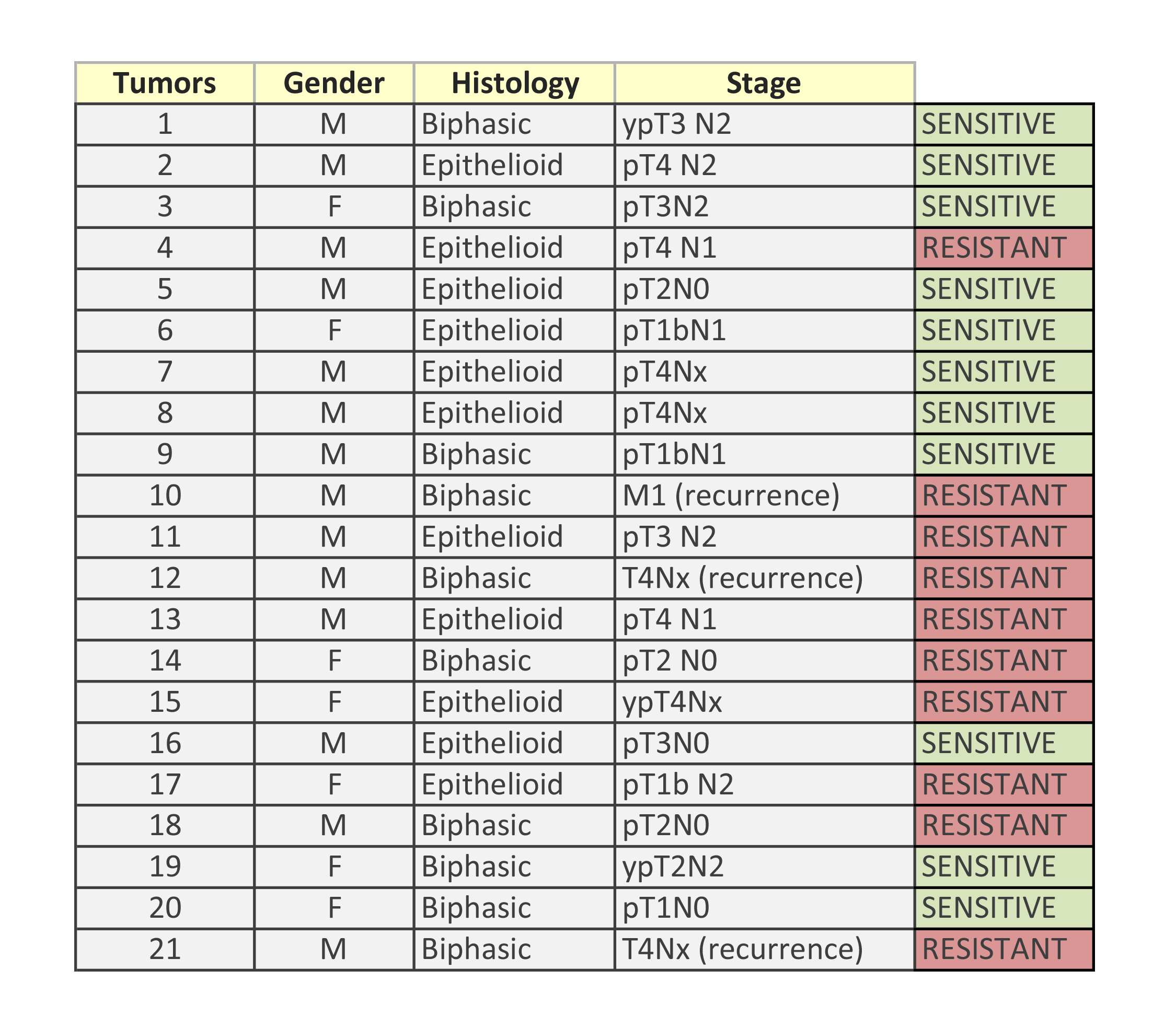

Supplement: S1 Table — Tumor fragment spheroids were grown from 11 epithelioid and 10 biphasic samples, with a range of tumor stages. Neither gender, histology nor stage was clearly associated with a response to GDC-0980. (TIF) [file pone.0134825.s008.tif]
